# Long-term administration of morphine specifically alters the level of protein expression in different brain regions and affects the redox state

**DOI:** 10.1515/biol-2022-0858

**Published:** 2024-04-20

**Authors:** Lucie Hejnova, Anna Hronova, Zdenka Drastichova, Jiri Novotny

**Affiliations:** Department of Physiology, Faculty of Science, Charles University, Prague, Czech Republic

**Keywords:** morphine, withdrawal, brain, oxidative stress, proteomics

## Abstract

We investigated the changes in redox state and protein expression in selected parts of the rat brain induced by a 4 week administration of morphine (10 mg/kg/day). We found a significant reduction in lipid peroxidation that mostly persisted for 1 week after morphine withdrawal. Morphine treatment led to a significant increase in complex II in the cerebral cortex (Crt), which was accompanied by increased protein carbonylation, in contrast to the other brain regions studied. Glutathione levels were altered differently in the different brain regions after morphine treatment. Using label-free quantitative proteomic analysis, we found some specific changes in protein expression profiles in the Crt, hippocampus, striatum, and cerebellum on the day after morphine withdrawal and 1 week later. A common feature was the upregulation of anti-apoptotic proteins and dysregulation of the extracellular matrix. Our results indicate that the tested protocol of morphine administration has no significant toxic effect on the rat brain. On the contrary, it led to a decrease in lipid peroxidation and activation of anti-apoptotic proteins. Furthermore, our data suggest that long-term treatment with morphine acts specifically on different brain regions and that a 1 week drug withdrawal is not sufficient to normalize cellular redox state and protein levels.

## 1 Introduction

Morphine is an effective drug for pain relief. However, its use is problematic as it causes many undesirable side effects, particularly dependence. There are currently a growing number of publications dealing with the cytotoxic or protective effects of chronic morphine treatment [1,2,3,4]. The cytotoxic effects of morphine are sometimes attributed to an increase in oxidative stress, but the effect of morphine on the oxidative state of cells is still controversial. There is evidence that chronic morphine treatment causes long-term changes in the regulation of signaling pathways that may persist even after the drug is discontinued [5,6,7,8,9,10].

In animal models, chronic administration of morphine was found to increase oxidative stress in brain tissue. Increased lipid peroxidation and decreased levels of reduced glutathione (GSH) [11,12,13] and decreased activity of manganese superoxide dismutase (SOD) and glutathione peroxidase (GPx) were found [14,15]. Interestingly, in contrast to animal studies, no change in GSH levels has been found in autopsies of the brains of heroin or cocaine addicts [16]. On the contrary, some studies have described the cytoprotective effects of morphine. In particular, the cardioprotective effects of pharmacological morphine preconditioning have been demonstrated in models of myocardial ischemia [17,18,19]. The neuroprotective effects of opioid preconditioning have been observed in various brain injuries and diseases such as stroke, neonatal hypoxia/ischemia, trauma, Parkinson’s disease, and Alzheimer’s disease [20,21,22,23,24]. One of the parameters frequently monitored was the oxidative state of brain tissue. In some studies, chronic morphine alone did not cause an increase in lipid peroxidation and a decrease in catalase (CAT) activity, while on the other hand, a progressive increase in lipid peroxidation and a decrease in CAT activity after ischemia were counterbalanced by chronic morphine [22]. The activity of SOD was not influenced by chronic morphine, and conversely, prior chronic administration of morphine suppressed the decrease in SOD activity after ischemia [25]. The involvement of oxidative stress has been demonstrated in many neurodegenerative diseases such as Parkinson’s and Alzheimer’s [26,27]. Importantly, chronic administration of morphine or other opioids has been shown to have beneficial effects in some experimental models of neurodegenerative diseases. The reduction of oxidative stress is considered one of the possible mechanisms [28].

Cell membranes are an essential component of cells. The plasma membrane not only separates the internal milieu of the cell from the external environment, but various signals are also specifically targeted and transmitted through this indispensable cellular structure. The mitochondrial membranes house the respiratory chain, which is important for adenosine triphosphate formation. Damage to either membrane can have significant consequences for cell survival.

The effect of morphine administration was also investigated at the level of changes in protein levels by proteomic studies [29]. From this perspective, morphine has been shown to lead to a significant dysregulation of biological pathways associated with extracellular matrix organization, cell adhesion, cytoskeleton, energy metabolism, and glutamate homeostasis [9,30,31].

We have previously reported that the administration of morphine in a single dose (10 mg/kg) for 10 days may have cardioprotective effects by reducing the susceptibility of the heart to ischemia-induced ventricular arrhythmias [17]. Similar protective effects were observed after the prolonged administration of the drug and our proteomic analyses of left ventricular tissue revealed a number of changes in myocardial protein expression after morphine administration and withdrawal [32,33]. A constant dose of 10 mg/kg morphine per day was used in various studies in rats [34,35,36,37]. In addition, long-term treatment with such a relatively high dose of morphine corresponds to the situation of drug addicts who can ingest similar amounts of the drug [38]. In the present study, we decided to investigate the consequences of prolonged morphine administration and morphine withdrawal in selected parts of the rat brain (cortex [Crt], hippocampus [Hip], striatum [Str], and cerebellum [Crb]). As mentioned earlier, morphine can affect the redox balance and potentially cause oxidative stress. Our specific aims were therefore initially to determine the effect of morphine on the extent of oxidative damage to cell membranes prepared from brain tissue. In this context, we decided to investigate the effect of morphine on the amount of reduced GSH, changes in the expression of respiratory chain complexes and antioxidant enzymes. The second aim was to determine the effects of morphine and its withdrawal on the overall expression of brain proteins using proteomic analysis.

## 2 Methods

### 2.1 Materials

Morphine sulfate was obtained from Saneca Pharmaceuticals, Ltd. (Hlohovec, Slovakia). Some of the basic chemicals (Tris, sucrose, glycine, Tween 20) were from Serva (Heidelberg, Germany). All other chemicals were purchased from Sigma-Aldrich (St. Louis, MO, USA).

### 2.2 Morphine treatment

Male Wistar rats (weighing 250–280 g) were maintained on a 12/12 light/dark cycle and had ad libitum access to food and water. The rats were divided into three groups of six animals each. The rats in the morphine group (M28) received 10 mg/kg morphine dissolved in 0.9% NaCl daily for 28 days. A withdrawal group of rats (M28/W) received morphine at the same dose for 28 days, followed by 7 days without the drug. The control rats (CON) received 0.9% NaCl. The rats in the CON and M28 groups were sacrificed 24 h after the last injection, and the rats in the M28/W group after the next 7 days. Samples of Crt, Hip, Str, and Crb were collected immediately, snap-frozen in liquid nitrogen, and stored at −80°C until use.


**Ethical approval:** The research related to animal use has been complied with all the relevant national regulations and institutional policies for the care and use of animals and has been approved by the Ministry of Education, Youth and Sports of the Czech Republic 116 (Licence No. MSMT-1479/2019–6).

### 2.3 Sample preparation

The individual parts of the brain were pooled into experimental groups. The pooled samples of prefrontal Crt, Hip, Str, and Crb were homogenized in TMES buffer (20 mM Tris, 3 mM MgCl_2_, 1 mM ethylenediamine-tetraacetic acid (EDTA), and 0.25 M sucrose; pH 7.4) using a glass-Teflon homogenizer at 1,200 rpm for 2 min on ice [39]. About 50 µl of the 20% (w/v) homogenate was withdrawn and mixed 1:1 with 4% sodium deoxycholate (Sigma-Aldrich, St. Louis, MO, USA) and used for label-free protein quantification (MaxLFQ). Peptidase and phosphatase inhibitors (cOmplete, PhosSTOP, Roche Diagnostics, Basel, Switzerland) were immediately added to the remaining homogenates. The homogenates were clarified by centrifugation at 800 × *g* for 10 min at 4°C (Hettich Universal 320 R; Hettich, Germany). The resulting supernatants were centrifuged at 50,000 × *g* for 30 min at 4°C (Beckman Optima L-90K, rotor Ti50, Beckman, USA). The cytosolic fractions (supernatant) were kept, and the crude membrane fractions (pellet) were dissolved in TME buffer (20 mM Tris, 3 mM MgCl_2_, and 1 mM EDTA; pH 7.4). Both types of samples were aliquoted and rapidly frozen in liquid nitrogen. The protein content was measured by the bicinchonic acid method [39].

### 2.4 Lipid peroxidation

Lipid peroxidation was measured by malondialdehyde (MDA) formation using the thiobarbituric acid reactive substance (TBARS) assay [40] in crude membrane fractions. About 50 µg of protein diluted in 100 µl of TME was mixed with 200 µl of 10% trichloroacetic acid (TCA) and 300 µl of 0.67% thiobarbituric acid. The mixtures were heated at 100°C for 30 min. After cooling, the mixtures were centrifuged (Hettich Universal 320 R, Hettich, Germany) for 10 min at 10,000 rpm and 4°C. The supernatant (100 µl) was pipetted onto a 96-well plate, and the fluorescence intensity was read (*λ*_ex/em_ = 540/590 nm) using plate reader (BioTek Synergy HT, Winooski, VA, USA). The same procedure was used for the standards, and the amounts of TBARS in the samples were calculated.

### 2.5 Lipid content

Lipids were isolated from crude membrane fractions using the Folch method [41]. The total amount of lipids in the samples was detected using a colorimetric method in microtiter plate format [42]. In brief, 360 µg of proteins in 65 µl of TME was sonicated with 40% potency (Sonopuls, Bandelin electronic, Berlin, Germany) for 10 s, then mixed with 1.3 ml of chloroform:methanol (2:1), and sonicated one more time. The solutions were centrifuged at 620 × *g* for 20 min at room temperature (RT), and the lipid extract in the chloroform:methanol fraction was then evaporated at 90°C for 20 min. The lipids were resuspended in 450 µl of chloroform:methanol, and 100 µl of the extracts was pipetted onto a plate and evaporated under the same conditions. Then, 150 µl of concentrated H_2_SO_4_ was added and incubated at 90°C for 10 min. The solution (100 µl) was pipetted into a new plate, and the background absorbance was measured at 540 nm. Then, 100 µl of vanillin–phosphoric acid reagent (0.2 mg vanillin to 1 ml 17% phosphoric acid) was added and incubated for 15 min at RT in the dark. The absorbance of the sample was measured at 540 nm using plate reader (BioTek Synergy HT, Winooski, VA, USA). The total amount of lipids in the samples was calculated using Brain Extract Total (Sigma-Aldrich, St. Louis, MO, USA) as a standard.

### 2.6 Protein carbonylation

The oxidation of the proteins in the crude membrane fractions was measured as the amount of carbonyls using a simplified spectrophotometric 2,4-dinitrophenylhydrazine (DNPH) assay [43]. About 500 µg of protein diluted in 200 µl of TME was mixed with 200 µl of 10 mM DNPH and incubated for 10 min at RT in the dark. Then, 100 µl of 6 M NaOH was added, vortexed vigorously, and incubated for a further 10 min. Then, 100 µl of the mixture was pipetted into a 96-well plate, and the absorbance was measured at 450 nm using plate reader (BioTek Synergy HT, Winooski, VA, USA). The concentration of carbonyl groups was calculated using the molar extinction coefficient of DNPH at 450 nm (*ε* = 22,308 M^−1^ cm^−1^). DNPH was dissolved in dimethyl sulfoxide (DMSO) and diluted in 2.5 M HCl (the final concentration of DMSO was 1%).

### 2.7 Reduced GSH

The amount of reduced GSH in the cytosolic fractions was determined by the Ellman method [44]. Briefly, 600 µg of protein diluted in 200 µl of TME was mixed with 200 µl of 10% TCA and incubated on ice for 10 min. After incubation, the mixture was centrifuged (Hettich Universal 320 R, Hettich, Tuttlingen, Germany) at 10,000 rpm for 10 min at 4°C. The supernatant (100 µl) was pipetted onto a 96-well plate, and 100 µl of 0.5 M Na_2_HPO_4_ (pH 8.4) and 20 µl of 1 mM 5,5′-dithio-bis-[2-nitrobenzoic acid] (DTNB) in 1% sodium citrate were added. The solution was incubated at RT for 10 min. The GSH standard curve was prepared in the same way. The absorbance of the samples was determined at 405 nm using plate reader (BioTek Synergy HT, Winooski, VA, USA), and the amount of reduced GSH was calculated from the standard curve.

### 2.8 Westerm blotting

Samples of the crude membrane fraction were solubilized in Laemmli buffer and loaded onto standard 10% polyacrylamide gels as described previously [45]. Electrophoresis was performed at 200 V for 45 min. Subsequently, the resolved proteins were transferred to a nitrocellulose membrane (Protran BA85, GE Healthcare, Little Chalfont, Buckinghamshire, UK) at 100 V for 90 min, blocked with 5% nonfat dry milk in TBS buffer (10 mM Tris and 150 mM NaCl; pH 8.0) for 30 min, and then incubated with specific primary antibodies overnight at 4°C. The respiratory chain proteins were detected with Total OXPHOS Rodent WB Antibody Cocktail (Abcam, Cambridge, UK) and Complex II (CII) 70 kDa (Invitrogen, Carlsbad, California, USA). The next day, the membranes were washed three times for 10 min with TBS buffer containing 0.3% Tween 20 (TBS-T), and secondary anti-mouse IgG antibodies conjugated to horseradish peroxidase (Amersham, Little Chalfont, UK) were applied for 1 h at RT. After washing three times for 10 min with TBS-T, the blots were visualized by enhanced chemiluminescence technique using SuperSignal West Dura substrate (Pierce Biotechnology, Rockford, IL, USA). Nitrocellulose membranes were stained with Ponceau S dye to verify uniform loading of each sample. The blots were scanned and quantitatively analyzed using ImageJ software. The band intensity in the Western blot was normalized to the total protein content (Ponceau staining).

### 2.9 MaxLFQ

The samples prepared for label-free quantification were processed as previously described [9]. They were reduced with 5 mM Tis-(2-carboxyethyl)-phosphine for 60 min at 60°C, and free cysteine residues were blocked by 10 mM methyl methanethiosulfonate in isopropanol and 10 min incubation at RT. Trypsin digestion was performed by adding 4 µg of trypsin to each sample and incubating for 12 h at 37°C. Trypsin cleavage was terminated by acidifying the reaction mixture with trifluoroacetic acid to a final concentration of 0.1%. Sodium deoxycholate was removed by extraction with two volumes of ethyl acetate (8,000 × *g*, 30 s). The aqueous phase containing the peptide mixture was added to SpeedVac for 20 min, and the samples were desalted using MacroTrap C18 cartridge (MICHROM Bioresources, Inc., Auburn, CA, USA). Finally, 2 µg of the peptide mixture was loaded onto the PepMap 300 C 18 column (5 mm × 300 μm ID, 5 μm particles, 300 Å pore size; 163589, Thermo Scientific, Waltham, MA, USA). Individual peptides were separated by high pressure liquid chromatography using an EASY-Spray column, 50 cm × 75 μm ID, PepMap C18, 2 μm particles, 100 Å pore size (ES 803, Thermo Scientific, Waltham, MA, USA). Peptides were separated using a linear gradient for 2 h between mobile phase A (2% acetonitrile, 0.1% formic acid) and B (80% acetonitrile, 0.1% formic acid). At the beginning of the separation, the system was run with 2% mobile phase B, followed by gradient elution to 40% B. The eluted peptide cations were converted to gas-phase ions by electrospray ionization and analyzed using a Thermo Orbitrap fusion mass spectrometer (Q-OT-qIT, Thermo Scientific, Waltham, MA, USA). Survey scans of peptide precursors from 400 to 1600 *m*/*z* were performed with a resolution of 120 K (at 200 *m*/*z*) and an ion count of 5 × 10^5^. Tandem mass spectrometry was performed by isolation at 1.5 Th with quadrupole, HCD fragmentation with normalized collision energy of 30, and rapid scan MS analysis in the ion trap. The MS^2^ ion counting target was set to 10^4^, and the maximum injection time was 35 ms. Only the precursors with charge states 2–6 were sampled for MS^2^. The dynamic exclusion time was set to 45 s with a tolerance of 10 ppm for the selected precursor and its isotope. The monoisotopic precursor selection was turned on. The instrument was operated in maximum speed mode with 2-s cycles.

### 2.10 Data analysis

At least three determinations were carried out in various experiments. For some measurements, a higher number of replicates were used in order to achieve better reproducibility and reliability of the results. The results were statistically analyzed using two-way ANOVA with Tukey’s multiple comparison test, and *P* values of less than 0.05 were considered significant. GraphPad Prism version 8 software (GraphPad Software Inc., La Jolla, CA, USA) was used for statistical analysis and graph generation. Proteomic data were analyzed and quantified using MaxQuant software (version 1.5.2.4., Planck Institute of Biochemistry, Munich, Germany). The false discovery rate was set to 1% for both proteins and peptides, and a minimum length of seven amino acids was specified. The Andromeda search engine was used to search MS/MS spectra against the UniProt *Rattus norvegicus* database of protein sequences. Enzyme specificity was set as C-terminal to Arg and Lys, with cleavage at proline bonds and a maximum of two failed cleavages also allowed. Dithiomethylation of cysteine was selected as fixed modification and N-terminal protein acetylation and methionine oxidation as variable modifications. The “match between runs” feature of MaxQuant was used to transfer identifications to other LC–MS/MS runs based on their masses and retention times (maximum deviation 0.7 min) to other LC–MS/MS runs; this was also used in quantification experiments. Quantifications were performed using the recently described label-free algorithms [46]. Binary logarithms of intensity ratios were then calculated for each group, and the difference between control and sample means were determined. Only the minimum 2-fold differences were considered, which were calculated for at least two readings from three replicates. Altered proteins were analyzed using the DAVID database for GO terms biological processes and cellular components [47]. Significant clusters of proteins with more than four or ten proteins (biological processes and cellular components, respectively) were considered.

## 3 Results

### 3.1 Oxidative damage to membranes

**Figure 1:**
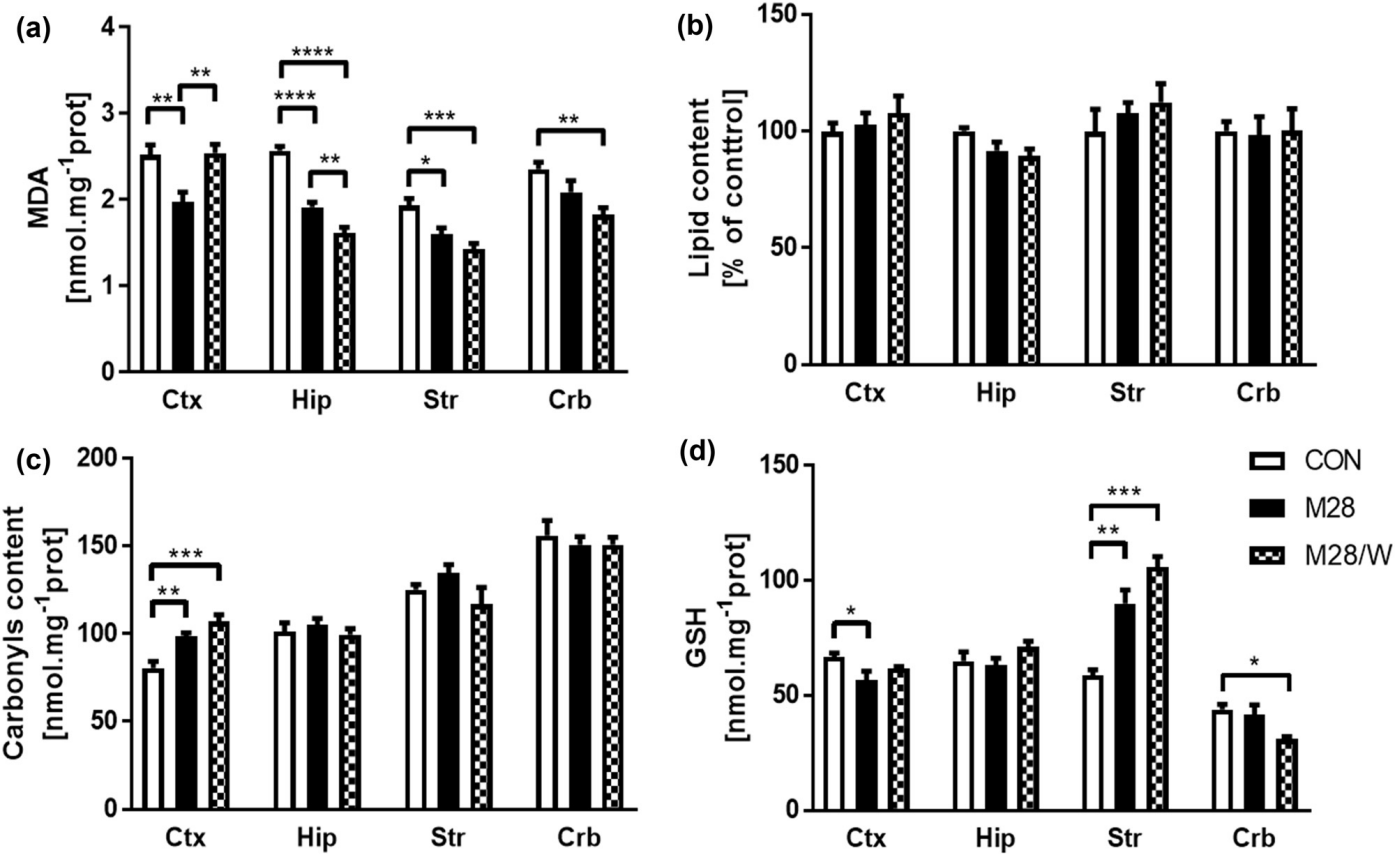
Oxidative damage of cell membranes. Lipid peroxidation (a), total lipid content (b), protein carbonylation (c), and reduced GSH (d) were measured as described in the methods. Bars represent the mean ± standard error of the mean (SEM). Lipid peroxidation was measured as MDA formation six times in Crt and Str, five times in Hip and Crb, all measurement was done in tetraplicates. Protein carbonylation was determined five times in triplicate. Reduced GSH was measured four times in triplicate. Total lipid content was measured five times (striatum four times) in tetraplikates. Bars represent mean ± SEM (* *p* ≤ 0.05, ** *p* ≤ 0.01, *** *p* ≤ 0.001, **** *p* ≤ 0.0001).

Chronic administration of morphine at a dose of 10 mg/kg/day for 28 days and subsequent withdrawal resulted in significantly decreased lipid peroxidation in samples of crude membranes prepared from all brain parts tested (Figure 1a). In the Crt, lipid peroxidation, as determined by measuring MDA production, was significantly reduced in animals killed immediately after 28 days of morphine administration (M28). However, after 7 days of withdrawal (M28/W), MDA levels returned to control values (CON). In the Str and Hip, MDA levels were significantly decreased after morphine administration (M28), and this decrease was exacerbated after the 7 day withdrawal (M28/W). A decreased amount of MDA was also detectable in the Crb, but the reduction was only significant in the RM28 group compared to the control group (CON). Total lipid levels in the crude membrane fraction were not altered by morphine treatment and withdrawal (Figure 1b).

Damage to the membrane and membrane-associated proteins was determined by measuring protein carbonylation (Figure 1c). The results showed that with the exception of the Crt, there was no change in protein carbonylation after morphine withdrawal. In the Crt of the M28 and M28/W groups, protein carbonylation increased by 10 and 15%, respectively. Interestingly, the level of protein carbonylation differed significantly between brain regions. Protein carbonylation increased in the following order: Crt < Hip < Str < Crb (Figure 1c).

The different parts of the rat brain also differed in the amount of reduced GSH, another parameter of redox balance (Figure 1d). Crt and Hip contained similar amounts of GSH in the control condition, while the content of this tripeptide was lower in the Str and significantly lower in the Crb. GSH content was significantly decreased in the Crt of M28 rats (by 10%) and in the Crb of M28/W rats (by 20%) compared to the corresponding controls. On the other hand, the GSH content in the Hip was not significantly altered after morphine withdrawal. In contrast, the GSH content was markedly increased in the Str of M28 (by 25%) and M28/W (by 35%) rats.

### 3.2 Respiratory chain

**Figure 2:**
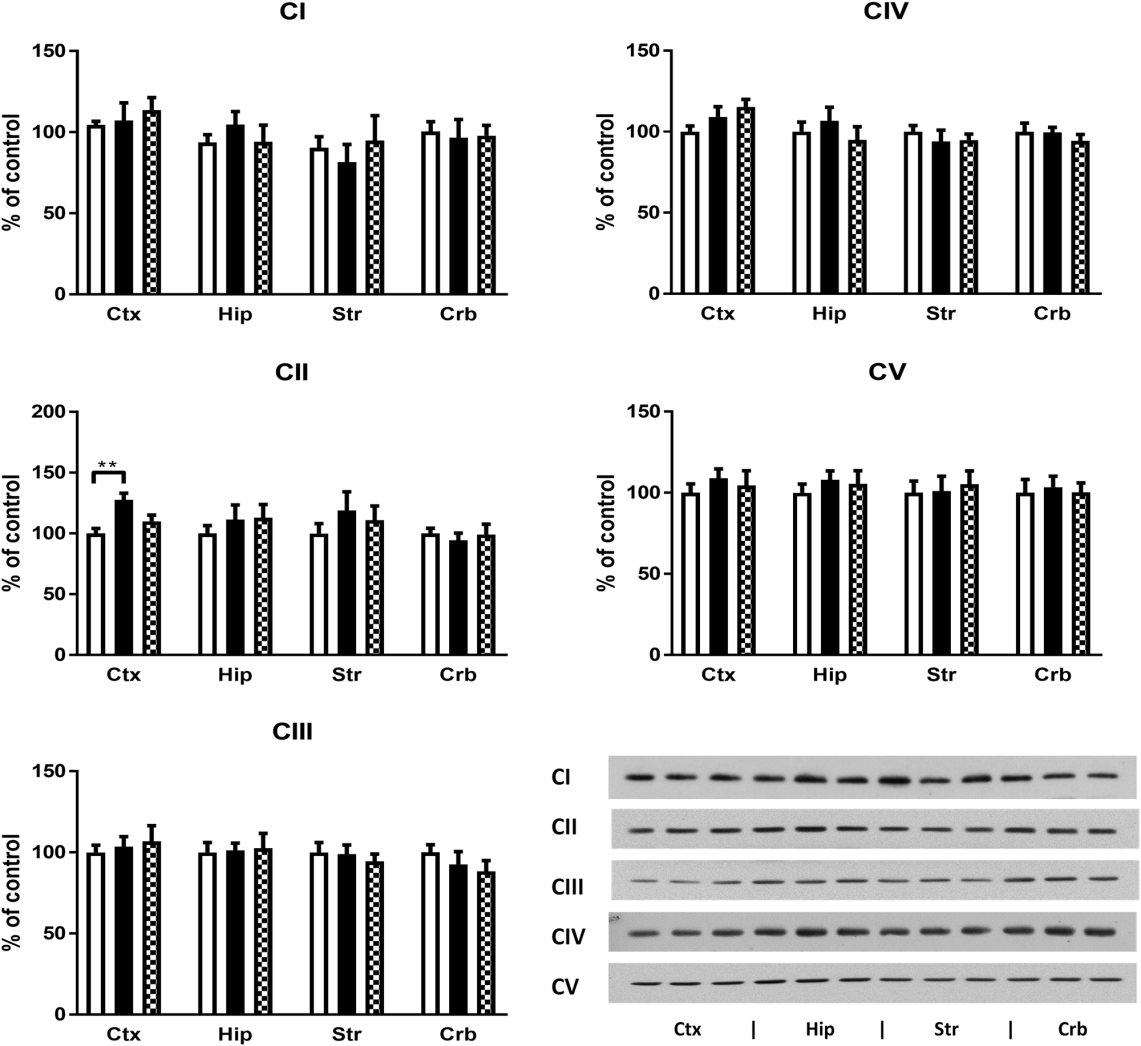
Effect of morphine on the respiratory chain. Individual complexes (CI–CV) of the respiratory chain in samples of the Crt, Hip, Str, and Crb were assessed by Western blotting using Total OXPHOS Antibody Cocktail and specific anti-CII antibody. Bars represent the mean ± SEM from six separate experiments (** *p* ≤ 0.01).

The proteins of the respiratory chain were analyzed by Western blotting with the Total OXPHOS Antibody Cocktail. CII was detected separately with a specific antibody. In most cases, there were no significant differences between the groups. The only change was found in the Crt, where a significantly increased amount of CII was observed in the M28 group (Figure 2).

### 3.3 Free protein quantification

**Figure 3:**
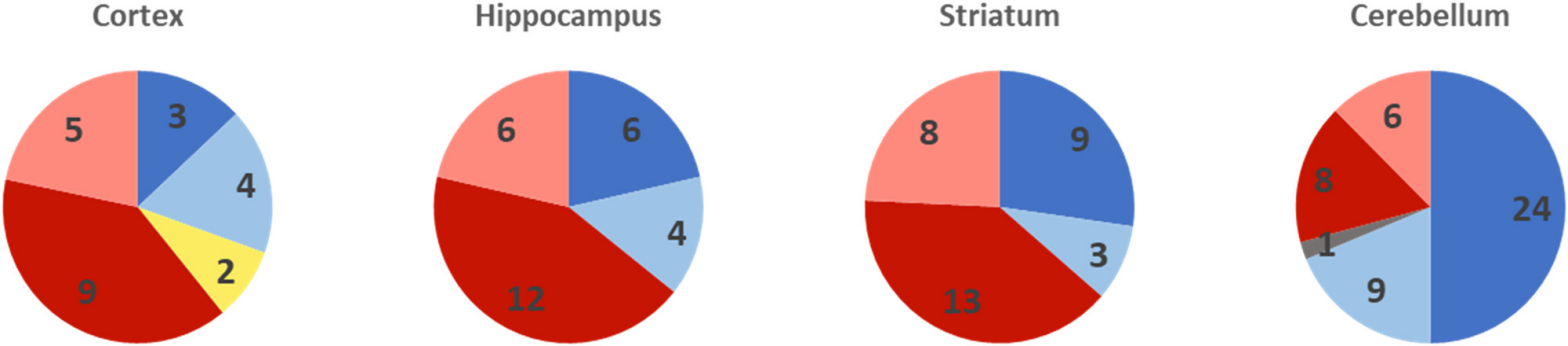
Total number of changed proteins in individual parts of the rat brain after morphine treatment. Dark blue – proteins upregulated in M28 and/or RM28, light blue – proteins detected in M28 and/or RM28, grey – proteins upregulated in M28 and downregulated in RM28, yellow – proteins downregulated in M28 and upregulated in RM28, dark red – proteins downregulated in M28 and/or RM28, light red – proteins not detected in M28 and/or RM28.

Using LFQ, we determined 2,463 proteins in the Crt, 2,458 proteins in the Hip, 2,448 proteins in the Str, and 2,416 proteins in the Crb. The detected redox enzymes were not affected by morphine and withdrawal in any part of the brain (Table 1). However, various changes in the levels of some proteins (Table S1) and qualitative changes in the proteins were observed (Table S2). In general, a relatively equal number of proteins were upregulated or downregulated in all parts of the brain except the Crb. Obviously, increased levels were found for more proteins in the Crb than in other parts of the brain (Figure 3). The altered proteins were analyzed using the DAVID database. Based on the analysis of biological processes and cellular components (Table S3), several clusters of altered proteins were found. The analysis of biological processes revealed a protein cluster in all brain regions, representing the negative regulation of apoptotic processes. Another protein cluster was detected in the Str and seven other proteins in the Crb. Analysis of the cellular components of all parts of the brain revealed a cluster of proteins of extracellular exosomes, except in the Crt. No protein cluster was found in the cerebral Crt according to the selected parameters. Another protein cluster was detected in the Str. A total of seven protein clusters were distinguished in the Crb.

**Table 1:** Redox enzymes not affected by morphine

Protein ID	Protein name	Gene name
P04762	CAT	Cat
P07632	SOD [Cu–Zn]	Sod1
P07895	SOD [Mn], mitochondrial	Sod2
P29476	Nitric oxide synthase, brain	Nos1
P04041	Glutathione peroxidase 1	Gpx1
Q63716	Peroxiredoxin-1	Prdx1
P35704	Peroxiredoxin-2	Prdx2
Q9Z0V6	Thioredoxin-dependent peroxide reductase, mitochondrial	Prdx3
Q9Z0V5	Peroxiredoxin-4	Prdx4
Q9R063	Peroxiredoxin-5, mitochondrial	Prdx5
O35244	Peroxiredoxin-6	Prdx6

The expression of various proteins was altered in a similar way in all parts of the brain examined. Morphine treatment significantly decreased the levels of flotillin-1 and flotillin-2, and this effect was enhanced after 7 days of withdrawal. Another downregulated protein was metabotropic glutamate receptor 2 (mGLUR2). The level of this receptor was reduced in all parts of the brain except the Crb, and the decrease persisted after discontinuation of the drug. In the Crb, the level of mGLUR2 in the M28 group was below the detection limit of the method. Some proteins were upregulated in all parts of the brain. The 70 kDa heat shock protein (Hsp70) (1 A and 1B detected together) was increased in the M28 group. After the discontinuation of morphine (M28/W), the level of this protein decreased but remained significantly above the control value. Heat shock protein beta-1 (Hspb1) was also upregulated by morphine treatment. Higher levels of this protein were measured in the Hip, Str, and Crb in both experimental groups. The enzyme protein-arginine deiminase type-2 (PAD2) was upregulated in all parts of the brain, and the increased levels remained the same in the M28 and M28/W groups. Phospholipase C-delta1 (PLCdelta1) is another protein that was elevated in all parts of the brain. This protein was not detected in the Crt, Hip, and Crb of the control animals, but after morphine treatment, its levels rose above the detection limit. Only in the Str was PLCdelta1 detected in all experimental groups, and its levels were increased in both the M28 and RM28 groups. The change in PLCdelta1 expression after the 7 day withdrawal was no more than 2-fold different from that in the M28 group. Only in a few cases did protein levels return to control levels after morphine discontinuation (e.g., collagen type VI alpha 1 chain in the Hip and NADH-ubiquinone oxidoreductase chain 4, heat shock protein beta-1 and endoplasmic reticulum membrane protein complex subunit 8 in Str, sorbin and SH3 domain-containing protein 2, erythrocyte membrane protein band 4.1, filamin A, anion exchange protein, erythrocyte membrane protein band 4.2, tropomyosin alpha-1 chain, vimentin, alpha-crystallin B chain, and neurabin-1 in the Crb). On the other hand, the effect of morphine on some proteins was enhanced after discontinuation of the drug (e.g., myosin regulatory light chain 2 and myosin-6 in Crt, and myosin-6 in the Hip). Exceptionally, protein expression was opposite in the M28 and M28/W groups (e.g., collagen alpha-1(I) chain and collagen alpha-2(I) chain in the Crt, myelin protein P0 in the Crb), or protein expression was altered only in the M28/W group (e.g., myosin light chain 3 and GPRIN family member 3 in the Crt, Thy-1 membrane glycoprotein, mitochondrial glutamate carrier 2, inter alpha-trypsin inhibitor heavy chain 4, keratin type I cytoskeletal 18, and keratin type II cytoskeletal 8 in the Hip, cAMP Regulated Phosphoprotein 21 (ARPP-21), cytochrome c oxidase subunit 6A1, plasminogen, serine proteinase inhibitor clade A member 3 C, alpha-1-antiproteinase, fibronectin, fetuin-B, and vitamin D-binding protein in the Str and zinc transporter 3 in the Crb) (Table S1). The same qualitative changes were found for four proteins in all parts of the brain examined. A version of cytochrome c oxidase subunit 2 (CcO2) and sideroflexin-3 were detected in M28 and M28/W, whereas an isoform of reticulon and an isoform of microtubule-associated protein 1A (MAP1A) were detected only in the control rats and not in the morphine-affected animals (M28 and M28/W). In parallel, similar qualitative changes were observed in a number of other proteins (Table S2).

## 4 Discussion

One of the main objectives of this study was to investigate the consequences of prolonged morphine administration and subsequent drug discontinuation on the redox state in the cerebral Crt, Hip, Str, and Crb of rats, as well as the possible effect of morphine on cell membrane damage determined by the detection of lipid peroxidation and protein carbonylation in selected brain regions. The extent of lipid peroxidation and protein carbonylation are commonly used markers of possible oxidative damage. In parallel, GSH levels were monitored in the same brain tissue samples. We observed some specific changes in some of the brain parts examined. Interestingly, plasma and mitochondrial membrane proteins were significantly oxidatively damaged only in the Crt. This damage persisted even after 7 days of morphine withdrawal. No changes in protein carbonylation were detected in other parts of the brain. This is partly in contrast to the results of a study by Sumathi et al., who observed increased protein carbonylation in rat whole brain homogenates after 21 days of morphine administration [14]. However, these authors used much higher doses of morphine (10–160 mg/kg/day) and did not differentiate between individual parts of the brain.

Whether chronic morphine use can cause oxidative brain damage remains controversial. There are essentially two main types of studies that have addressed this question. Some studies have demonstrated oxidative damage to the brain from chronic morphine use and have looked for substances that could mitigate this adverse effect. Conversely, other studies have not observed morphine-induced oxidative stress in the brain and have aimed to evaluate the potential protective effects of morphine against ischemia or neurodegenerative processes. There are two research groups investigating oxidative damage caused by chronic morphine. Motaghinejad et al. used a model based on the treatment of rats with morphine at a dose of 45 mg/kg/day for 4 weeks [15,48]. The same dosing regimen was used in a similar study by another research group [4]. In these studies, an increase in MDA and a decrease in the total amount of reduced GSH in the brain were observed. Similar results were obtained by Abdel-Zaher et al. in a mouse model and administration of morphine for 7 days at a dose of 5 mg/kg twice daily [11,12,13]. In contrast, the administration of morphine at a dose of 5 mg/kg/day for 6 consecutive days did not result in a change in MDA and a reduction in GSH in the brain of mice [49]. In rats, different effects were observed when 1 and 5 mg/kg/day were administered for 15 days. The low dose of morphine did not lead to an increase in MDA levels, in contrast to a higher dose [50]. Specific schedules of morphine administration led to different results in lipid peroxidation and GSH in rat brain [48]. Interestingly, contradictory results were found in the autopsied brains of chronic heroin users. Either an unchanged amount of GSH [16] or a decrease in GSH was found [51]. In contrast to the aforementioned observations, chronic administration of morphine alone did not result in an increase in lipid peroxidation in studies addressing the protective effects of morphine against oxidative stress. Chronic administration of morphine resulted in a decrease in lipid peroxidation after *in vivo* induced ischemia [22,52] or after the addition of H_2_O_2_ to cells in *in vitro* models [53]. The protocol of morphine administration appears to be an important factor influencing MDA production. The present results are broadly consistent with this knowledge. Our regimen of morphine administration resulted in a significant decrease in lipid peroxidation. This decrease persisted after 7 days of morphine withdrawal, except in the cerebral Crt, where MDA levels returned to control values. The decrease in lipid peroxidation cannot be explained by the mere reduction in total lipids, as the total lipid content was not changed. We were particularly interested in the lipids of the mitochondrial and plasma membrane. Obviously, cells must effectively protect these membranes from potential oxidative damage, so lipid peroxidation in these structures was reduced accordingly. A decrease in GSH in the cytosol was only observed in the Crt and Crb. In contrast, the amount of GSH was increased in the Str. Morphine has been shown to alter dopamine neurotransmission in the Str [54,55]. Interestingly, it has been reported that increased presynaptic dopamine turnover leads to the formation of deaminated metabolites and increased levels of oxidized GSH in the Str, but not in the frontal Crt [56]. Oral administration of GSH reduced infarct size in the cerebral ischemia/reperfusion model in rats by promoting the synthesis and inhibiting the degradation of intrastriatal dopamine. Administration of dopamine precursors had a similar effect on infarct size [57]. Our label-free determination of protein expression revealed an increased level of aromatic-l-amino-acid decarboxylase (P14173) in the Str. This may be suggested that the modification of dopamine neurotransmission by morphine could increase GSH levels in the Str.

Oxidative stress is caused by an increased production of reactive oxygen species (ROS). An important source of ROS in the cell is the respiratory chain [58]. Therefore, determining the expression of respiratory chain complexes was part of our main objective. Here, we observed a significant increase in the amount of CII in the Crt after morphine treatment. This increase may indicate an increased production of ROS by morphine in the Crt [59]. The increased production of ROS in the Crt of the M28 group could explain the increased carbonylation of proteins that was only detected in the Crt. Interestingly, the time course of morphine-induced changes in lipid peroxidation was also different in the Crt and in other parts of the brain studied. Although MDA formation in the Crt was reduced in the M28 group similarly to other parts of the brain, it returned to control levels 1 week after drug discontinuation, in contrast to the other parts of the brain. The reduced MDA formation observed in the brains of morphine-treated rats suggests an antioxidant effect of this drug. On the other hand, the increased expression of CII observed in the cerebral Crt after morphine administration could be associated with increased ROS production and the initiation of some compensatory mechanisms, which, in turn, could temporarily suppress MDA formation. These results indicate a greater vulnerability of the prefrontal Crt to the effects of morphine. In terms of addiction development, this result could imply further development of addiction by enhancing compulsive drug use and weakening volitional control [60].

The cellular redox state is also regulated by some enzymes that reduce ROS and thus act as antioxidant agents. Chronic administration of morphine has been shown to reduce the activity of some of these enzymes. The activity of SOD and GPx was decreased after 4 weeks of administration of high doses (45 mg/kg/day) of morphine [15,4]. In contrast, no decrease in the activities of these enzymes was observed in the studies dealing with morphine preconditioning. On the other hand, chronic administration of morphine had beneficial effects on the activity of antioxidant enzymes after ischemia. Ischemia significantly reduced enzyme activity, and morphine preconditioning suppressed this reduction [22,25]. For these reasons, we focused on the detection of these proteins to achieve our main goal. Our protocol of morphine administration (10 mg/kg/day, 28 days) did not result in changes in the expression of enzymes (CAT, SOD [Cu–Zn], mitochondrial SOD [Mn], brain nitric oxide synthase, GPx, peroxiredoxin-1,2,4,5,6, and mitochondrial thioredoxin-dependent peroxide reductase) that affect the cellular redox state (Table 1).

In the second aim of our study, we assessed the changes in protein expression determined by label-free protein quantification. Our data are consistent with other studies in which more proteins were downregulated than upregulated in the brain after morphine treatment [61,30].

The expression levels of some proteins were similarly altered in the different brain regions. The levels of two heat shock proteins (Hsp70 and Hspb1) were elevated. Similar results were reported for Hsp70 at both protein [50,62,63] and mRNA levels [64,65,66] and also for Hspb1 [66]. Both heat shock proteins are induced by a variety of noxious stimuli, such as ischemia, epileptic seizures, and hyperthermia, and confer protection to cells [67,68,69]. Elevated levels of Hsp70 protect against oxidative stress by reducing lipid peroxidation and increasing the activity of some enzymes that regulate cellular redox state [70,71]. It can be speculated that although the expression levels of redox enzymes were not altered, the long-term (28 days) upregulation of Hsp70 did not increase their activity and may contribute to a reduction in oxidative stress. The second question is whether upregulated Hsp70 could reduce the lipid peroxidation observed in our study. Ischemic postconditioning in the kidney reduced the adverse effects of ischemia such as lipid peroxidation, apoptosis, and inflammation by increasing the expression of Hsp70 and Hspb1 [72]. Another upregulated protein was PAD2. This enzyme catalyzes the conversion of arginine to citrulline. Its substrates include vimentin, myelin basic protein, and also histones. Increased activity of this enzyme has been found to be associated with multiple sclerosis, but also with Alzheimer’s disease [73]. Lange et al. reported that the inhibition of PAD2 reduced the extent of damage after a hypoxic–ischemic insult in neonates [74]. The question arises whether PAD2 expression increased by morphine contributes more to oxidative cell damage or whether it is related to gene transcription by histone modification [75]. The last increased protein detected in all parts of the brain was PLCdelta1. PLCdelta1 has been found to accumulate in filamentous inclusions in human neurodegenerative diseases [76]. The expression of PLCdelta1 has been shown to be increased by oxidative stress. However, the changes in the overall activity of PLCdelta1 are unclear [77,78]. The activity of PLCdelta1 may also be regulated by Gi/o proteins due to Ca^2+^ mobilization in cells [79]. Morphine treatment reduced the levels of flotillin-1 and flotillin-2, both of which are scaffold proteins associated with rafts. Their interactions enable the formation of multiprotein complexes and are therefore involved in a variety of cellular events from cell signaling, endocytosis, and protein trafficking to gene expression [80]. The link between flotillin and oxidative stress is related to neuroglobin, the protein that is cytoprotective in many ways [81]. One of these is the binding to flotillin-1 during oxidative stress and its function as a guanine nucleotide dissociation inhibitor for Gαi/o. This interaction is neuroprotective [82]. The link between neuroglobin, flotillin-1, and the Gi/o protein may be impaired by the administration of morphine, which acts via Gi/o proteins. Chronic morphine treatment under our experimental conditions resulted in a decrease in the expression of flotillin-1, but neuroglobin was not detected. Reduced expression of flotillin could affect the function of proteins associated with them, not only neuroglobin. Another downregulated protein was mGLUR2. Activation of mGLUR2 protects cells from oxidative stress damage after hypoxia–ischemia [83]. Under our experimental conditions, a decrease in mGLUR2 expression was observed in the M28 group and this decrease was more pronounced after withdrawal (RM28). Downregulation of mGLUR2 after morphine withdrawal has already been demonstrated in the *nucleus accumbens*, where this decrease in mGLUR2 expression abolished long-term depression [84]. Transgenic mGluR2-knockout rats showed a stronger dopamine response to heroin administration in the *nucleus accumbens*, higher locomotor activity, and stronger morphine analgesia. Thus, mGLUR2 has antiopioid effects [85,86]. The observed decrease in mGLUR2 expression could be responsible for the increasing dependence.

Interestingly, the protein expression profiles of all brain areas examined after 7 days of morphine withdrawal (M28/W) did not differ significantly from those of the M28 group. Only a few proteins were significantly altered. These results indicate that a 1 week morphine withdrawal does not lead to a normalization of protein levels and a return to the pre-dependence state. The common qualitative changes involved four proteins. CcO2 is part of complex IV of the respiratory chain. Only the CcO2 version Q5UAJ6 was detected in all parts of the brain of the M28 and RM28 groups. The other CcO2 detected did not change. This result is consistent with Western blot results, which did not show any significant changes in complex IV. The expression of the CcO2 version could be related to the inhibitory effect of morphine on cytochrome C oxidase activity. Morphine activates the production of NO [87], which subsequently inhibits cytochrome C oxidase [88]. Another protein that was only detected in M28 and RM28 was sideroflexin-3, which transports serine into the mitochondria. This is an important step in one-carbon metabolism [89]. 1C units of one-carbon C metabolism also serve to form NADPH, which is important for cell redox homeostasis [90,91]. The upregulation of sideroflexin-3 could increase NADPH production. Reticulon was not detected after morphine treatment and 7 day withdrawal. Reticulons are membrane proteins that are localized in the endoplasmic reticulum and have various functions [92]. MAP1A is preferentially localized in the dendrites of adult neurons, where it stabilizes microtubules [93]. The observed downregulation of MAP1A may partially reduce synaptic transduction. MAP1A-knockout mice showed the destabilization of dendritic structure, synaptic plasticity, and NMDA trafficking [94]. In the Crb, disruption of the MAP1A gene resulted in abnormal focal swelling of dendrite shafts and disruption of axon initial segment morphology [95].

Our analysis of the altered proteins using the DAVID database with respect to biological processes revealed a cluster of proteins that negatively regulate apoptotic processes in each part of the brain tested (Table S3). This group of proteins includes, in particular, Hsp70, Hspb1, and Nucleolar protein 3 (Nol3). The expression of all these proteins was increased. Hsp70, Hspb1, and Nol3 can inhibit apoptosis through numerous interactions with multiple mechanisms such as inhibition of caspases 3 and 9, inhibition of Bad and Bax, prevention of Bcl2 degradation, and inhibition of the JNK signaling pathway of apoptosis [96,97,98,99]. Hsp70 is involved in many processes, and its crucial role in neurodegeneration has also been described [100]. Increased Hspb1 expression may protect neurons after lumbosacral nerve root avulsion. This effect is due to the suppression of oxidative stress responses, as demonstrated in the SH-SY5Y model of benign and primary neuronal cultures exposed to oxygen–glucose deprivation [101]. A protective effect was also demonstrated in the cardiomyocyte line H9c2 after exposure to oxidative stress by H_2_O_2_ [102]. The protection of neuronal HT22 cells against oxidative stress induced by H_2_O_2_ administration was mediated by the regulation of apoptotic signaling pathways by added Nol3. Transduced Nol3 also protected against neuronal death in the CA1 region of the Hip in a model of ischemic injury [103]. Analysis of the data by cellular components revealed changes in extracellular space in the Hip, Str, and Crb (Table S4). The levels of most structural proteins related to extracellular space and exosome formation were reduced by morphine and its withdrawal in the Hip and Str, but also in the Crt, where the analysis did not identify this cluster due to the small number of protein changes (e.g., collagen alpha-1(I) chain, collagen alpha-2(I) chain, collagen type VI alpha 1 chain, flotillin-2, flotillin-1, fibronectin, reticulon). Similarly, cytoskeletal proteins and proteins that influence its composition and function were also reduced (e.g., spectrin, alpha, erythrocytic 1, MAP1A, myosin-6). A completely different situation was observed in the Crb. In this part of the brain, morphine induced an upregulation of structural proteins of the extracellular matrix and the cytoskeleton (e.g., collagen alpha-1(I) chain, collagen alpha-2(I) chain, collagen type VI alpha 1 chain, prolargin, fibronectin, biglycan, spectrin, alpha, erythrocytic 1, actin, alpha cardiac muscle 1, sorbin and SH3 domain-containing protein 2, erythrocyte membrane protein band 4.1). This difference is also evident in Fiure 3 and in the number of seven protein clusters detected by DAVID analysis focusing on cellular components (Table S4). In general, morphine administration leads to structural changes in the architecture of the brain. Our study shows that the Crb is most affected by structural changes. The observed upregulation of the extracellular matrix, cytoskeleton, and cytoskeleton-associated proteins in the Crb correlates well with morphological changes described previously [104]. Studies focusing on the effect of opioids on the extracellular matrix are not yet numerous [105]. Another approach to study changes in the extracellular matrix is to observe changes in extracellular vesicles. Morphine has caused changes in the composition of extracellular vesicles released by human brain microvascular endothelial cells. Pathway enrichment analysis revealed the “cell adhesion and extracellular matrix remodeling” process and the “HIF1 pathway”, a pathway related to oxidative stress responses [106]. Morphine-induced changes in the extracellular matrix were also detected by proteomic and phosphoproteomic analyses of proteins in a model of primary human astrocytes using liquid chromatography–electrospray ionization-mass spectrometry [31]. A 7 day morphine withdrawal showed a variety of protein changes. The levels of some proteins returned to control values or even fluctuated in the other direction. For some proteins, the effect was even more pronounced and changes were observed in other proteins that had not changed immediately after morphine withdrawal [9,10].

## 5 Conclusion

This study demonstrates that long-term treatment of rats with moderate doses of morphine leads to specific changes in the protein expression profile of different brain regions that do not return to normal after 7 days of drug withdrawal. In general, the monitored biochemical parameters and protein expression were not fundamentally altered. Only four qualitative changes in proteins common to all parts of the brain studied (cytochrome C oxidase subunit 2, sideroflexin-3, reticulon, and MAP1A) were detected in brain samples from rats withdrawn from morphine for 1  or 7 days compared to controls. Interestingly, glutathione levels were altered differently in the different brain regions and the observed significant increase in CII in the Crt was accompanied by increased protein carbonylation in this part of the brain, in contrast to the other brain regions studied. Our data suggest that long-term treatment with morphine specifically affects different brain regions and that a 1 week withdrawal of the drug is not sufficient to normalize protein levels and the cellular redox state. The data obtained expand our understanding of the potential adverse effects of morphine on the proteome and redox state of the brain and support the notion that morphine dosage plays an important role in the potential oxidative damage to cell membranes in the brain. A certain limitation of the study is that effects of morphine were only tested in male rats and that only a 1 week withdrawal period after discontinuation of the drug was investigated. Future studies looking at the long-term effects of morphine on the brain should also include female animals and longer withdrawal periods.

## Supplementary Material

supplementary material
