# Supplementary material for: Long-term administration of morphine specifically alters the level of protein expression in different brain regions and affects the redox state
Source: Open Life Sci. 2024 Apr 20;19(1):20220858. doi: 10.1515/biol-2022-0858 (PMC11049758; doi:10.1515/biol-2022-0858)
Supplement: supplementary material [file biol-2022-0858-sm.pdf]

Supplementary material

Table S1: Changed proteins detected using label-free quantification

Protein ID	Protein name	Gene name	M28 vs CON	*p* value	M28/W vs CON	*p* value	Function
**Cortex**							
P0DMW0 P0DMW1	Heat shock 70 kDa protein 1A and 1B	Hspa1a Hspa1b	7.2	<0.0001	3.5[a]	<0.0001	Molecular chaperones
Q62881	Nucleolar protein 3	Nol3	2.3	0.0020	2.6	0.0087	Apoptosis repressor that blocks multiple modes of cell death.
P20717	Protein-arginine deiminase type-2	Padi2	2.2	0.0003	2.0	0.0005	Catalyzes the deimination of arginine residues of proteins
P16409	Myosin light chain 3	Myl3	—	—	−2.3	0.0073	The regulatory light chain of myosin. Does not bind calcium.
D3ZF21	GPRIN family member 3	Gprin3	—	—	−2.8	0.0106	May be involved in neurite outgrowth.
G3V784	ADP-dependent glucokinase	Adpgk	−2.0	0.0003	−2.1	0.0020	Catalyzes the phosphorylation of D-glucose to D-glucose 6-phosphate using ADP as the phosphate donor
P31421	Metabotropic glutamate receptor 2	Grm2	−2.4	0.0003	−3.4	0.0041	G-protein coupled receptor for glutamate
P02454	Collagen alpha-1(I) chain	Col1a1	−2.8	0.0007	2.6[a]	<0.0001	The main structural protein in the extracellular matrix
P02466	Collagen alpha-2(I) chain	Col1a2	−2.9	0.0027	2.0[a]	0.0035	The main structural protein in the extracellular matrix
P02767	Transthyretin	Ttr	−3.0	0.0014	−2.6	0.0005	Thyroid hormone-binding protein
P08733	Myosin regulatory light chain 2	Myl2	−3.2	0.0004	ND[a]		The regulatory protein of myosin
Q9Z2S9	Flotillin-2	Flot2	−4.6	<0.0001	−4.6	0.0001	Integral membrane protein associated with caveolae
Q9Z1E1	Flotillin-1	Flot1	−5.1	<0.0001	−6.2	<0.0001	Integral membrane protein associated with caveolae
P02563	Myosin-6	Myh6	−7.0	0.0002	−711.9[a]	<0.0001	Motor protein
**Hippocampus**							
P0DMW0 P0DMW1	Heat shock 70 kDa protein 1A and 1B	Hspa1aHspa1b	4.5	<0.0001	2.3[a]	<0.0001	Molecular chaperones
P20717	Protein-arginine deiminase type-2	Padi2	2.8	0.0003	2.3	<0.0001	Catalyzes the deimination of arginine residues of proteins
P42930	Heat shock protein beta-1	Hspb1	2.7	0.0180	2.3	0.0225	Molecular chaperone
D3ZRN3	Actin, beta-like 2	Actbl2	2.3	Nonsig.	2.2	0.0248	Cytoskeleton
H1UBM8	Copine 4 protein	Cpne4	2.0	0.0024	2.0	0.0007	Calcium-dependent membrane-binding protein
P01830	Thy-1 membrane glycoprotein	Thy1	—	—	2.1[a]	0.0002	

(Continued)

Table S1: *Continued*

Protein ID	Protein name	Gene name	M28 vs CON	*p* value	M28/W vs CON	*p* value	Function
							May play a role in cell-cell or cell-ligand interactions during synaptogenesis and other events in the brain
Q505J6	Mitochondrial glutamate carrier 2	Slc25a18	—	—	−2.2	0.0081	Involved in the transport of glutamate across the inner mitochondrial membrane
O35802	Inter alpha-trypsin inhibitor, heavy chain 4	ITIH4	—	—	−2.0	0.0047	Involved in inflammatory responses to trauma
Q5BJY9	Keratin, type I cytoskeletal 18	Krt18	—	—	−2.1	0.0001	Cytoskeleton. play a role in filament reorganization
Q10758	Keratin, type II cytoskeletal 8	Krt8	—	—	−2.4	0.0027	Cytoskeleton
P31421	Metabotropic glutamate receptor 2	Grm2	−2.1	0.0050	−3.6	0.0040	G-protein coupled receptor for glutamate
P02454	Collagen alpha-1(I) chain	Col1a1	−2.6	0.0001	−2.8	0.0002	The main structural protein in the extracellular matrix
D3ZUL3	Collagen type VI alpha 1 chain	Col6a1	−2.8	0.0391	—	—	Collagen VI acts as a cell-binding protein
P02767	Transthyretin	Ttr	−3.1	0.0024	−2.7	0.0001	Thyroid hormone-binding protein
Q9Z1E1	Flotillin-1	Flot1	−4.1	<0.0001	−5.4	<0.0001	Integral membrane protein associated with caveolae
Q9Z2S9	Flotillin-2	Flot2	−4.5	<0.0001	−4.6	<0.0001	Integral membrane protein associated with caveolae
P02563	Myosin-6	Myh6	−32.6	0.0001	ND[a]		Motor protein
Q29RW1	Myosin-4	Myh4	−39.7	0.0002	−62.7	<0.0001	Motor protein
**Striatum**							
P0DMW0 P0DMW1	Heat shock 70 kDa protein 1A and 1B	Hspa1a Hspa1b	5.8	<0.0001	2.7[a]	<0.0001	Molecular chaperones
Q06QG7	NADH-ubiquinone oxidoreductase chain 4	Nd4	2.9	0.0244	—	—	Core subunit of the mitochondrial membrane respiratory chain NADH dehydrogenase (Complex I)
P14173	Aromatic-ʟ-amino-acid decarboxylase	Ddc	2.5	0.0067	2.5	0.0005	Catalyzes the decarboxylation of L-3,4-dihydroxyphenylalanine (DOPA) to dopamine, L-5-hydroxytryptophan to serotonin and ʟ-tryptophan to tryptamine
P42930	Heat shock protein beta-1	Hspb1	2.3	0.0072	—	—	Molecular chaperone
P20717	Protein-arginine deiminase type-2	Padi2	2.2	0.0013	2.3	0.0005	Catalyzes the deimination of arginine residues of proteins
P10688	Phospholipase C-delta-1	Plcd1	2.1	0.0195	2.4	0.0131	

(Continued)

Table S1: *Continued*

Protein ID	Protein name	Gene name	M28 vs CON	*p* value	M28/W vs CON	*p* value	Function
							The production of the second messenger molecules diacylglycerol and inositol 1,4,5-trisphosphate
Q5FVL2	ER membrane protein complex subunit 8	Emc8	2.0	0.0269	—	—	Membrane protein of endoplasmic reticulum
Q5FVI0	Arpp-21 protein	Arpp21	—	—	3.0	<0.0001	May act as a competitive inhibitor of calmodulin-dependent enzymes such as calcineurin in neurons
P10818	Cytochrome c oxidase subunit 6A1	Cox6a1	—	—	2.1	0.0027	Component of the cytochrome c oxidase. the last enzyme in the mitochondrial electron transport chain which drives oxidative phosphorylation
Q01177	Plasminogen	Plg	—	—	−2.1	0.0003	Zymogen of plasmin. Plasmin dissolves the fibrin of blood clots and acts as a proteolytic factor in a variety of other processes
A0A0G2JSK1	Serine proteinase inhibitor, clade A, member 3C	Serpina3c	—	—	−2.3	0.0005	Serine (or cysteine) peptidase inhibitor
P17475	Alpha-1-antiproteinase	Serpina1	—	—	−2.3	0.0001	Inhibitor of serine proteases
P04937	Fibronectin	Fn1	—	—	−3.1	0.0359	Involved in cell adhesion, cell motility, opsonisation, wound healing, and maintenance of cell shape
Q9QX79	Fetuin-B	Fetub	—	—	−3.1	0.0001	Cysteine protease inhibitors
P04276	Vitamin D-binding protein	Gc	—	—	−2.0	0.0027	Involved in vitamin D transport and storage
P26644	Beta-2-glycoprotein 1	Apoh	-2.1	0.0034	−2.6	0.0002	May prevent activation of the intrinsic blood coagulation cascade by binding to phospholipids on the surface of damaged cells
Q9WV48	SH3 and multiple ankyrin repeat domains protein 1	Shank1	−2.1	0.0055	−3.1	0.0007	Adapter protein in the postsynaptic density (PSD) of excitatory synapses
P24090	Alpha-2-HS-glycoprotein	Ahsg	−2.4	<0.0001	−2.5	<0.0001	Involved in several functions such as endocytosis, brain development and the formation of bone tissue
P31421	Metabotropic glutamate receptor 2	Grm2	−2.9	0.0060	−3.0	0.0365	G-protein coupled receptor for glutamate
Q9Z2S9	Flotillin-2	Flot2	−3.4	<0.0001	−4.5	<0.0001	Integral membrane protein associated with caveolae
Q9Z1E1	Flotillin-1	Flot1	−4.1	<0.0001	−6.2	<0.0001	Integral membrane protein associated with caveolae
D4A678	Spectrin, alpha, erythrocytic 1	Spta1	−5.2	0.0393	−7.5	0.0370	Major constituent of the cytoskeletal network

(Continued)

Table S1: *Continued*

Protein ID	Protein name	Gene name	M28 vs CON	*p* value	M28/W vs CON	*p* value	Function
**Cerebellum**							
D3ZUL3	Collagen type VI alpha 1 chain	Col6a1	16.6	0.0009	9.4	0.0014	Collagen VI acts as a cell-binding protein
P0DMW0 P0DMW1	Heat shock 70 kDa protein 1A and 1B	Hspa1a Hspa1b	10.0	<0.0001	4.7[a]	<0.0001	Molecular chaperones
D4A678	Spectrin, alpha, erythrocytic 1	Spta1	9.3	<0.0001	2.2[a]	0.0014	Major constituent of the cytoskeletal network
Q9EQP5	Prolargin	Prelp	8.9	<0.0001	3.5[a]	0.0061	Anchoring basement membranes to the underlying connective tissue
Q9WVJ6	Tissue-type transglutaminase	Tgm2	7.5	0.0002	3.0[a]	0.0017	Catalyses the cross-linking of proteins
Q923Z2	Tropomyosin 1. alpha	Tpm1	5.8	<0.0001	2.1[a]	0.0035	Actin-binding protein involved in the contractile system of striated and smooth muscles and the cytoskeleton of non-muscle cells
P02454	Collagen alpha-1(I) chain	Col1a1	5.7	<0.0001	9.2	<0.0001	The main structural protein in the extracellular matrix
P02466	Collagen alpha-2(I) chain	Col1a2	5.6	<0.0001	6.1	0.0001	The main structural protein in the extracellular matrix
Q63862	Myosin-11	Myh11	4.4	<0.0001	2.8	0.0001	Motor protein
P68035	Actin, alpha cardiac muscle 1	Actc1	4.3	<0.0001	2.1[a]	0.0004	Cytoskeleton
O35413	Sorbin and SH3 domain-containing protein 2	Sorbs2	4.3	0.0008	–[a]	—	Adapter protein that plays a role in the assembling of signaling complexes
P04937	Fibronectin	Fn1	4.1	<0.0001	3.4	<0.0001	Involved in cell adhesion. cell motility. opsonization. wound healing. and maintenance of cell shape
P62738 P63269	Actin, aortic smooth muscle	Acta2, Acta3	3.4	Nonsig.	2.2	0.0005	Cytoskeleton
D3ZIP3	Erythrocyte membrane protein band 4.1	Epb41	2.8	0.0279	—	—	The structural element of the membrane skeleton
P42930	Heat shock protein beta-1	Hspb1	2.8	0.0060	2.5	0.0114	Molecular chaperone
P06907	Myelin protein P0	Mpz P0	2.7	0.0004	−2.4[a]	0.0048	Adhesion molecule necessary for normal myelination
C0JPT7	Filamin A	Flna	2.6	<0.0001	—	—	Anchors various transmembrane proteins to the actin cytoskeleton and serves as a scaffold for a wide range of cytoplasmic signaling proteins
Q5XI86	Peptidyl-tRNA hydrolase 2	Ptrh2	2.6	0.0209	2.7	0.0102	Promotes caspase-independent apoptosis by regulating the function of two transcriptional regulators. AES and TLE1
P20717	Protein-arginine deiminase type-2	Padi2	2.5	0.0012	2.5	0.0008	Catalyses the deimination of arginine residues of proteins

(Continued)

Table S1: *Continued*

Protein ID	Protein name	Gene name	M28 vs CON	*p* value	M28/W vs CON	*p* value	Function
Q5U329	Anion exchange protein	Slc4a1	2.2	<0.0001	—	—	The transporter that mediates electroneutral anion exchange across the cell membrane
F1MAN8	Laminin subunit alpha 5	Lama5	2.2	0.0051	3.5	0.0010	Mediates the attachment. migration and organization of cells
B5DF57	Erythrocyte membrane protein band 4.2	Epb42	2.1	0.0371	—	—	The structural element of the membrane skeleton
P04692	Tropomyosin alpha-1 chain	Tpm1	2.1	0.0016	—	—	Actin-binding protein involved in the contractile system of striated and smooth muscles and the cytoskeleton of non-muscle cells
P31000	Vimentin	Vim	2.0	0.0001	—	—	Cytoskeleton
P23928	Alpha-crystallin B chain	Cryab	2.0	0.0003	—	—	Chaperone-like activity. preventing aggregation of various proteins under a wide range of stress conditions
Q6QIX3	Zinc transporter 3	Slc30a3	—	—	−2.8	0.0396	Involved in accumulation of zinc in synaptic vesicles
Q9QW07	Phospholipase C-beta-4	Plcb4	−2.0	Nonsig.	−2.3	0.0066	Production of the second messenger molecules diacylglycerol and inositol 1,4,5-trisphosphate
E9PTB2	Transcription elongation factor SPT5	Supt5h	−2.1	0.0003	−2.5	0.0019	Regulation of mRNA processing and transcription elongation by RNA polymerase II
Q01062	cGMP-dependent 3',5'-cyclic phosphodiesterase	Pde2a	−2.1	0.0236	−2.1	0.0416	Cyclic nucleotide phosphodiesterase with a dual-specificity for the second messengers cAMP and cGMP
O35867	Neurabin-1	Ppp1r9a	−2.7	0.0324	—	—	Binds to actin filaments (F-actin) and show cross-linking activity
Q9QUH6	Ras/Rap GTPase-activating protein SynGAP	Syngap1	−2.7	0.0093	−4.6	0.0002	The major constituent of the PSD, member of the NMDAR signaling complex in excitatory synapses
Q9Z1E1	Flotillin-1	Flot1	−4.2	<0.0001	−6.2	0.0001	Integral membrane protein associated with caveolae
Q9Z2S9	Flotillin-2	Flot2	−5.3	0.0001	−5.4	0.0014	Integral membrane protein associated with caveolae

ND, not detected; a, the difference in protein expression levels was more than 2× between the M28 and M28/W groups; –, unchanged protein expression between samples.

Table S2: Qualitative changes in protein expression

Protein ID	Protein name	Gene name	Detection	Function
**Cortex**				
Q5UAJ6	Cytochrome c oxidase subunit 2	COX2	M28, M28/W	Component of the cytochrome c oxidase. the last enzyme in the mitochondrial electron transport chain which drives oxidative phosphorylation
P10688	Phospholipase C-delta-1	Plcd1	M28, M28/W	The production of the second messenger molecules diacylglycerol and inositol 1,4,5-trisphosphate
Q9JHY2	Sideroflexin-3	Sfxn3	M28, M28/W	Mitochondrial serine transporter that mediates transport of serine into mitochondria
P06907	Myelin protein P0	Mpz	M28/W	Adhesion molecule necessary for normal myelination
Q8K5B5	Amino acid transporter	Slc1a2	CON	Sodium-dependent, high-affinity amino acid transporter that mediates the uptake of L-glutamate and also L-aspartate and D-aspartate
A0A0G2K1R5	CaM kinase-like vesicle-associated protein	Camkv	CON	Pseudokinase of the CaMK. synaptic protein crucial for dendritic spine maintenance
G3V7U2	Microtubule-associated protein 1A	Map1a	CON	Cytoskeleton
M0R608	Reticulon	Rtn1	CON	May be involved in neuroendocrine secretion or membrane trafficking in neuroendocrine cells
P63312	Thymosin beta-10	Tmsb10	CON, M28	Plays an important role in the organization of the cytoskeleton
**Hippocampus**				
M0RAD5	ATP-dependent Clp protease proteolytic subunit	Clpp	M28, M28/W	Hydrolysis of proteins to small peptides in the presence of ATP and magnesium
Q5UAJ6	Cytochrome c oxidase subunit 2	COX2	M28, M28/W	Component of the cytochrome c oxidase, the last enzyme in the mitochondrial electron transport chain which drives oxidative phosphorylation
P10688	Phospholipase C-delta-1	Plcd1	M28, M28/W	The production of the second messenger molecules diacylglycerol and inositol 1,4,5-trisphosphate
Q9JHY2	Sideroflexin-3	Sfxn3	M28, M28/W	Mitochondrial serine transporter that mediates transport of serine into mitochondria
A0A0G2K1R5	CaM kinase-like vesicle-associated protein	Camkv	CON	Pseudokinase of the CaMK. synaptic protein crucial for dendritic spine maintenance
G3V7U2	Microtubule-associated protein 1A	Map1a	CON	Cytoskeleton
G3V6E1	Myosin heavy chain 2	Myh2	CON	Motor protein
M0R608	Reticulon	Rtn1	CON	May be involved in neuroendocrine secretion or membrane trafficking in neuroendocrine cells
P05505	Cytochrome c oxidase subunit 3	Mtco3	CON, M28	Component of the cytochrome c oxidase, the last enzyme in the mitochondrial electron transport chain which drives oxidative phosphorylation
D4A2B0	DNA polymerase delta-interacting protein 3	Poldip3	CON, M28	Involved in regulation of translation
**Striatum**				
Q5UAJ6	Cytochrome c oxidase subunit 2	COX2	M28, M28/W	Component of the cytochrome c oxidase, the last enzyme in the mitochondrial electron transport chain which drives oxidative phosphorylation
Q62881	Nucleolar protein 3	Nol3	M28, M28/W	Apoptosis repressor that blocks multiple modes of cell death

(Continued)

Table S2: *Continued*

Protein ID	Protein name	Gene name	Detection	Function
Q9JHY2	Sideroflexin-3	Sfxn3	M28, M28/W	Mitochondrial serine transporter that mediates transport of serine into mitochondria
D4A817	Histone H2B	Hist2h2be	CON, M28/W	Core component of nucleosome
P63312	Thymosin beta-10	Tmsb10	CON, M28/W	Plays an important role in the organization of the cytoskeleton
A0A0G2K1R5	CaM kinase-like vesicle-associated protein	Camkv	CON	Pseudokinase of the CaMK. synaptic protein crucial for dendritic spine maintenance
P63219	Guanine nucleotide-binding protein G(I)/G(S)/G(O) subunit gamma-5	Gng5	CON	G protein signaling
D3ZVM5	Heat shock protein family A (Hsp70) member 12B	Hspa12b	CON	Chaperone protein
G3V7U2	Microtubule-associated protein 1A	Map1a	CON	Cytoskeleton
M0R608	Reticulon	Rtn1	CON	May be involved in neuroendocrine secretion or membrane trafficking in neuroendocrine cells
F1LRI5	eIF-2-alpha kinase activator GCN1	Gcn1	CON, M28/W	Acts as a positive activator of the EIF2AK4/GCN2 protein kinase activity
**Cerebellum**				
P47853	Biglycan	Bgn	M28, M28/W	May be involved in collagen fibre assembly
F1LNH3	Col6a2 protein	Col6a2	M28, M28/W	Collagen VI acts as a cell-binding protein
Q5UAJ6	Cytochrome c oxidase subunit 2	COX2	M28, M28/W	Component of the cytochrome c oxidase, the last enzyme in the mitochondrial electron transport chain which drives oxidative phosphorylation
P50609	Fibromodulin	Fmod	M28, M28/W	Affects the rate of fibrils formation
P16409	Myosin light chain 3	Myl3	M28, M28/W	The regulatory light chain of myosin. Does not bind calcium
Q64122	Myosin regulatory light polypeptide 9	Myl9	M28, M28/W	Myosin regulatory subunit that plays an important role in the regulation of both smooth muscle and nonmuscle cell contractile activity via its phosphorylation. Implicated in cytokinesis. receptor capping, and cell locomotion
P10688	Phospholipase C-delta-1	Plcd1	M28, M28/W	The production of the second messenger molecules diacylglycerol and inositol 1,4,5-trisphosphate
Q9JHY2	Sideroflexin-3	Sfxn3	M28, M28/W	Mitochondrial serine transporter that mediates transport of serine into mitochondria
Q6JAM9	Transmembrane protein 35A	Tmem35a	M28, M28/W	A soluble peptide released by shedding may interact with NGFR and modulate sympathetic neurite outgrowth
P62275	40S ribosomal protein S29	Rps29	CON, M28/W	Ribosome
P31421	Metabotropic glutamate receptor 2	Grm2	CON, M28/W	G-protein coupled receptor for glutamate
Q63942	GTP-binding protein Rab-3D	Rab3d	CON	Protein transport. Probably involved in regulated exocytosis
G3V7U2	Microtubule-associated protein 1A	Map1a	CON	Cytoskeleton

(Continued)

Table S2: *Continued*

Protein ID	Protein name	Gene name	Detection	Function
M0R608	Reticulon	Rtn1	CON	May be involved in neuroendocrine secretion or membrane trafficking in neuroendocrine cells
Q71DI1	Dermcidin	Dcd	CON, M28/W	Survival-promoting peptide promotes survival of neurons and displays phosphatase activity. It may bind IgG

Table S3: David analysis - Biological processes

Cluster	*p*-value	Proteins		
		**Protein ID**	**Protein name**	**Gene name**
**Cortex**				
Negative regulation of apoptotic process	0.0260	P0DMW0 P0DMW1	Heat shock 70 kDa protein 1A and 1B	Hspa1a Hspa1b
		P06907	Myelin protein P0	Mpz
		Q62881	Nucleolar protein 3	Nol3
**Hippocampus**				
Negative regulation of apoptotic process	0.0058	P0DMW0 P0DMW1	Heat shock 70 kDa protein 1A and 1B	Hspa1a Hspa1b
		P42930	Heat shock protein beta-1	Hspb1
		Q5BJY9	Keratin, type I cytoskeletal 18	Krt18
		P01830	Thy-1 membrane glycoprotein	Thy1
**Striatum**				
Negative regulation of apoptotic process	0.0110	P04937	Fibronectin	Fn1
		P0DMW0 P0DMW1	Heat shock 70 kDa protein 1A and 1B	Hspa1a Hspa1b
		P42930	Heat shock protein beta-1	Hspb1
		Q62881	Nucleolar protein 3	Nol3
Response to ischemia	<0.0001	P04937	Fibronectin	Fn1
		P0DMW0 P0DMW1	Heat shock 70 kDa protein 1A and 1B	Hspa1a Hspa1b
		P42930	Heat shock protein beta-1	Hspb1
		Q62881	Nucleolar protein 3	Nol3
**Cerebellum**				
Negative regulation of apoptotic process	0.0003	P68035	Actin, alpha cardiac muscle 1	Actc1
		P23928	Alpha-crystallin B chain	Cryab
		P04937	Fibronectin	Fn1
		C0JPT7	Filamin A	Flna
		P0DMW0 P0DMW1	Heat shock 70 kDa protein 1A and 1B	Hspa1a Hspa1b
		P42930	Heat shock protein beta-1	Hspb1
		P06907	Myelin protein P0	Mpz P0
Protein heterodimerization	<0.0001	P02454	Collagen alpha-1(I) chain	Col1a1
		P02466	Collagen alpha-2(I) chain	Col1a2
		D3ZUL3	Collagen type VI alpha 1 chain	Col6a1
		F1LNH3	Col6a2 protein	Col6a2
Response to ischemia	0.0002	P0DMW0 P0DMW1	Heat shock 70 kDa protein 1A and 1B	Hspa1a Hspa1b
		P42930	Heat shock protein beta-1	Hspb1
		P04937	Fibronectin	Fn1
Positive regulation of gene expression	0.0004	P68035	Actin, alpha cardiac muscle 1	Actc1
		P62738 P63269	Actin, aortic smooth muscle	Acta2, Acta3
		Q01062	cGMP-dependent 3',5'-cyclic phosphodiesterase	Pde2a
		P04937	Fibronectin	Fn1
		P0DMW0 P0DMW1	Heat shock 70 kDa protein 1A and 1B	Hspa1a Hspa1b
		P31000	Vimentin	Vim

(Continued)

Table S3: *Continued*

Cluster	*p*-value	Proteins		
		**Protein ID**	**Protein name**	**Gene name**
Wound healing	0.0041	P02454	Collagen alpha-1(I) chain	Col1a1
		P50609	Fibromodulin	Fmod
		P04937	Fibronectin	Fn1
		P04692	Tropomyosin alpha-1 chain	Tpm1
Protein stabilisation	0.0045	C0JPT7	Filamin A	Flna
		Q9Z2S9	Flotillin-2	Flot2
		P0DMW0 P0DMW1	Heat shock 70 kDa protein 1A and 1B	Hspa1a Hspa1b
Cell adhesion	0.0290	P04937	Fibronectin	Fn1
		Q9Z2S9	Flotillin-2	Flot2
		F1MAN8	Laminin subunit alpha 5	Lama5
		O35413	Sorbin and SH3 domain-containing protein 2	Sorbs2
Aging	0.0470	P23928	Alpha-crystallin B chain	Cryab
		P42930	Heat shock protein beta-1	Hspb1
		O35867	Neurabin-1	Ppp1r9a
		P31000	Vimentin	Vim

Table S4: David analysis - Cellular components

Cluster	*p*-value	Proteins		
		**Protein ID**	**Protein name**	**Gene name**
**Hippocampus**				
Extracellular exosome	<0.0001	D3ZRN3	Actin, beta-like 2	Actbl2
		D3ZUL3	Collagen type VI alpha 1 chain	Col6a1
		H1UBM8	Copine 4 protein	Cpne4
		Q5UAJ6	Cytochrome c oxidase subunit 2	COX2
		Q9Z1E1	Flotillin-1	Flot1
		Q9Z2S9	Flotillin-2	Flot2
		P42930	Heat shock protein beta-1	Hspb1
		O35802	Inter alpha-trypsin inhibitor, heavy chain 4	ITIH4
		Q5BJY9	Keratin, type I cytoskeletal 18	Krt18
		Q10758	Keratin, type II cytoskeletal 8	Krt8
		P10688	Phospholipase C-delta-1	Plcd1
		P20717	Protein-arginine deiminase type-2	Padi2
		P01830	Thy-1 membrane glycoprotein	Thy1
		P02767	Transthyretin	Ttr
**Striatum**				
Extracellular exosome	<0.0001	P17475	Alpha-1-antiproteinase	Serpina1
		P24090	Alpha-2-HS-glycoprotein	Ahsg
		P14173	Aromatic-L-amino-acid decarboxylase	Ddc
		P26644	Beta-2-glycoprotein 1	Apoh
		Q5UAJ6	Cytochrome c oxidase subunit 2	COX2
		Q9QX79	Fetuin-B	Fetub
		P04937	Fibronectin	Fn1
		Q9Z1E1	Flotillin-1	Flot1
		Q9Z2S9	Flotillin-2	Flot2
		P63219	Guanine nucleotide-binding protein G(I)/G(S)/G(O) subunit gamma-5	Gng5
		P42930	Heat shock protein beta-1	Hspb1
		P10688	Phospholipase C-delta-1	Plcd1
		P20717	Protein-arginine deiminase type-2	Padi2
		Q01177	Plasminogen	Plg
		P04276	Vitamin D-binding protein	Gc
Extracellular space	0.0002	P17475	Alpha-1-antiproteinase	Serpina1
		P24090	Alpha-2-HS-glycoprotein	Ahsg
		P26644	Beta-2-glycoprotein 1	Apoh
		Q9QX79	Fetuin-B	Fetub
		P04937	Fibronectin	Fn1
		P42930	Heat shock protein beta-1	Hspb1
		D4A817	Histone H2B	Hist2h2be
		Q01177	Plasminogen	Plg
		A0A0G2JSK1	Serine proteinase inhibitor, clade A, member 3C	Serpina3c

(Continued)

Table S4: *Continued*

Cluster	*p*-value	Proteins		
		**Protein ID**	**Protein name**	**Gene name**
		P04276	Vitamin D-binding protein	Gc
**Cerebellum**				
Extracellular exosome	<0.0001	P62275	40S ribosomal protein S29	Rps29
		P68035	Actin, alpha cardiac muscle 1	Actc1
		P62738 P63269	Actin, aortic smooth muscle	Acta2, Acta3
		P23928	Alpha-crystallin B chain	Cryab
		Q5U329	Anion exchange protein	Slc4a1
		P47853	Biglycan	Bgn
		P02466	Collagen alpha-2(I) chain	Col1a2
		D3ZUL3	Collagen type VI alpha 1 chain	Col6a1
		F1LNH3	Col6a2 protein	Col6a2
		Q5UAJ6	Cytochrome c oxidase subunit 2	COX2
		P04937	Fibronectin	Fn1
		C0JPT7	Filamin A	Flna
		Q9Z1E1	Flotillin-1	Flot1
		Q9Z2S9	Flotillin-2	Flot2
		P42930	Heat shock protein beta-1	Hspb1
		F1MAN8	Laminin subunit alpha 5	Lama5
		Q63862	Myosin-11	Myh11
		P10688	Phospholipase C-delta-1	Plcd1
		Q9EQP5	Prolargin	Prelp
		P20717	Protein-arginine deiminase type-2	Padi2
		Q9WVJ6	Tissue-type transglutaminase	Tgm2
		P31000	Vimentin	Vim
Extracellular space	0.0039	P68035	Actin, alpha cardiac muscle 1	Actc1
		P62738 P63269	Actin, aortic smooth muscle	Acta2, Acta3
		P02454	Collagen alpha-1(I) chain	Col1a1
		P02466	Collagen alpha-2(I) chain	Col1a2
		F1LNH3	Col6a2 protein	Col6a2
		P50609	Fibromodulin	Fmod
		P04937	Fibronectin	Fn1
		P42930	Heat shock protein beta-1	Hspb1
		F1MAN8	Laminin subunit alpha 5	Lama5
		Q9EQP5	Prolargin	Prelp
Extracellular matrix	<0.0001	P47853	Biglycan	Bgn
		P02454	Collagen alpha-1(I) chain	Col1a1
		P02466	Collagen alpha-2(I) chain	Col1a2
		D3ZUL3	Collagen type VI alpha 1 chain	Col6a1
		F1LNH3	Col6a2 protein	Col6a2

(Continued)

Table S4: *Continued*

Cluster	*p*-value	Proteins		
		**Protein ID**	**Protein name**	**Gene name**
		P50609	Fibromodulin	Fmod
		P04937	Fibronectin	Fn1
		C0JPT7	Filamin A	Flna
		P42930	Heat shock protein beta-1	Hspb1
		F1MAN8	Laminin subunit alpha 5	Lama5
		Q9EQP5	Prolargin	Prelp
		Q9WVJ6	Tissue-type transglutaminase	Tgm2
		P31000	Vimentin	Vim
Focal adhesion	<0.0001	P62275	40S ribosomal protein S29	Rps29
		P68035	Actin, alpha cardiac muscle 1	Actc1
		C0JPT7	Filamin A	Flna
		Q9Z1E1	Flotillin-1	Flot1
		Q9Z2S9	Flotillin-2	Flot2
		P0DMW0 P0DMW1	Heat shock 70 kDa protein 1A and 1B	Hspa1a Hspa1b
		P42930	Heat shock protein beta-1	Hspb1
		O35413	Sorbin and SH3 domain-containing protein 2	Sorbs2
		Q9WVJ6	Tissue-type transglutaminase	Tgm2
		P31000	Vimentin	Vim
Cytosol	0.0042	P23928	Alpha-crystallin B chain	Cryab
		Q01062	cGMP-dependent 3',5'-cyclic phosphodiesterase	Pde2a
		C0JPT7	Filamin A	Flna
		P0DMW0 P0DMW1	Heat shock 70 kDa protein 1A and 1B	Hspa1a Hspa1b
		G3V7U2	Microtubule-associated protein 1A	Map1a
		O35867	Neurabin-1	Ppp1r9a
		Q5XI86	Peptidyl-tRNA hydrolase 2	Ptrh2
		P10688	Phospholipase C-delta-1	Plcd1
		Q9WVJ6	Tissue-type transglutaminase	Tgm2
		P31000	Vimentin	Vim
Membrane	0.0052	P68035	Actin, alpha cardiac muscle 1	Actc1
		Q5U329	Anion exchange protein	Slc4a1
		D3ZUL3	Collagen type VI alpha 1 chain	Col6a1
		Q5UAJ6	Cytochrome c oxidase subunit 2	COX2
		D3ZIP3	Erythrocyte membrane protein band 4.1	Epb41
		D3ZIP3	Erythrocyte membrane protein band 4.2	Epb42
		C0JPT7	Filamin A	Flna
		Q9Z1E1	Flotillin-1	Flot1
		Q9Z2S9	Flotillin-2	Flot2
		Q5XI86	Peptidyl-tRNA hydrolase 2	Ptrh2
		P10688	Phospholipase C-delta-1	Plcd1

(Continued)

Table S4: *Continued*

Cluster	*p*-value	Proteins		
		**Protein ID**	**Protein name**	**Gene name**
		D4A678	Spectrin, alpha, erythrocytic 1	Spta1
		Q9QUH6	Ras/Rap GTPase-activating protein SynGAP	Syngap1
Cytoplasm	0.0150	P62275	40S ribosomal protein S29	Rps29
		P68035	Actin, alpha cardiac muscle 1	Actc1
		P62738 P63269	Actin, aortic smooth muscle	Acta2, Acta3
		P23928	Alpha-crystallin B chain	Cryab
		P47853	Biglycan	Bgn
		Q01062	cGMP-dependent 3',5'-cyclic phosphodiesterase	Pde2a
		P02454	Collagen alpha-1(I) chain	Col1a1
		D3ZIP3	Erythrocyte membrane protein band 4.1	Epb41
		C0JPT7	Filamin A	Flna
		P0DMW0 P0DMW1	Heat shock 70 kDa protein 1A and 1B	Hspa1a Hspa1b
		P42930	Heat shock protein beta-1	Hspb1
		G3V7U2	Microtubule-associated protein 1A	Map1a
		Q9QW07	Phospholipase C-beta-4	Plcb4
		P10688	Phospholipase C-delta-1	Plcd1
		P20717	Protein-arginine deiminase type-2	Padi2
		Q9QUH6	Ras/Rap GTPase-activating protein SynGAP	Syngap1
		Q9WVJ6	Tissue-type transglutaminase	Tgm2
		P04692	Tropomyosin alpha-1 chain	Tpm1
		P31000	Vimentin	Vim
		Q6QIX3	Zinc transporter 3	Slc30a3

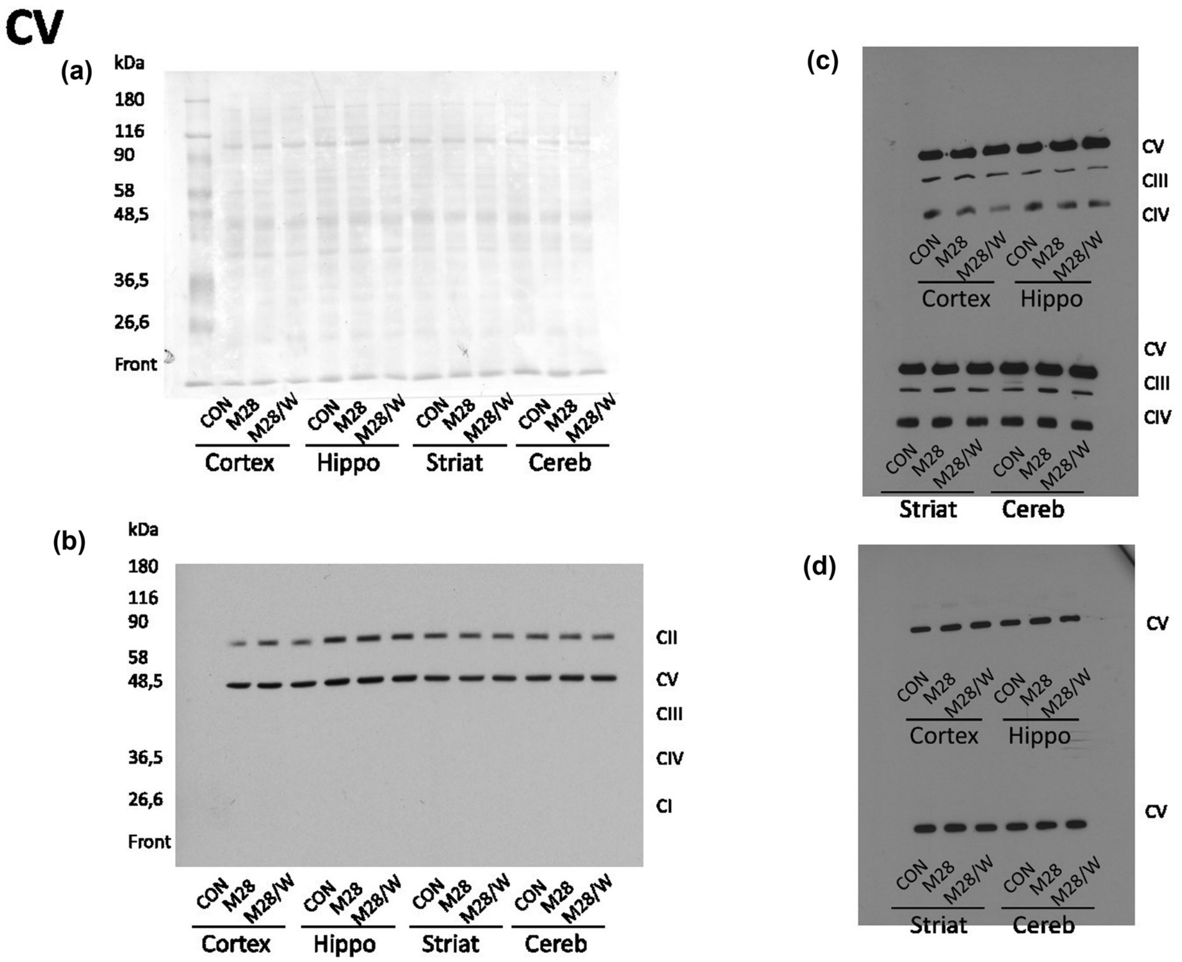

Figure S1: Typical Ponceau S staining and Western blots for detection of complex V. Proteins (10 µg protein per lane) in samples from cortex, hippocampus (Hippo), striatum (Striat) and cerebellum (Cereb) were separated using SDS-PAGE (10% gel, at constant 200 V for 45 min.) and transferred to a nitrocellulose membrane (at constant voltage 100 V for 90 min.). The total amount of proteins bound to the membranes was stained with Ponceau S (a). Complex V of the respiratory chain was detected with Total OXPHOS Rodent WB antibody diluted 1:200,000 (a) or 1:50,000 (c), and (d).

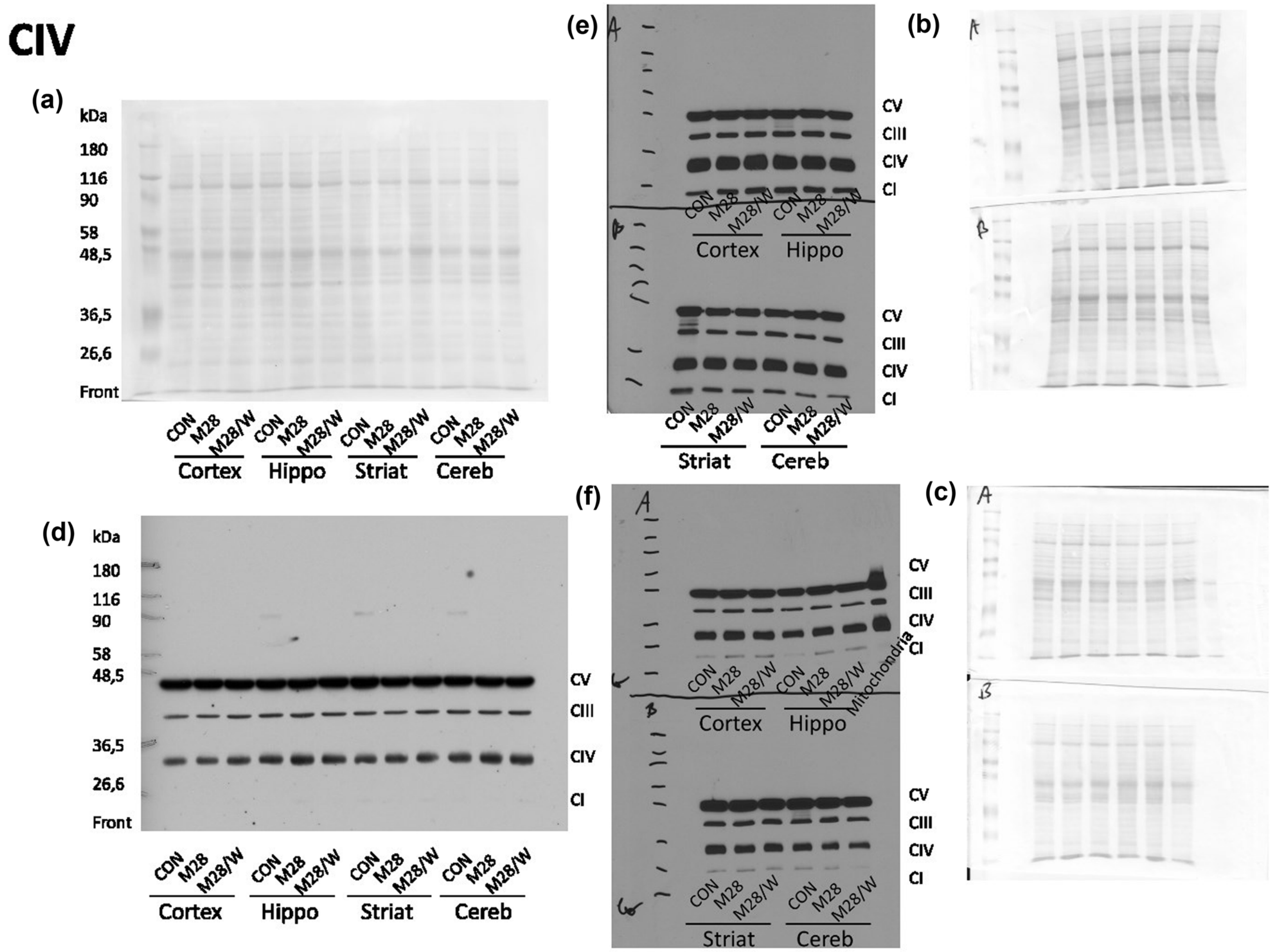

Figure S2: Typical Ponceau S staining and Western blots for detection of complex IV. Proteins (10 µg protein per lane) in samples from cortex, hippocampus (Hippo), striatum (Striat) and cerebellum (Cereb) were separated using SDS-PAGE (10 % gel, at constant 200 V for 45 min.) and transferred to a nitrocellulose membrane (at constant voltage 100 V for 90 min.). The total amount of proteins bound to the membranes was stained with Ponceau S (a)–(c). Complex IV of the respiratory chain was detected with Total OXPHOS Rodent WB antibody diluted 1:50,000 (d)–(f).

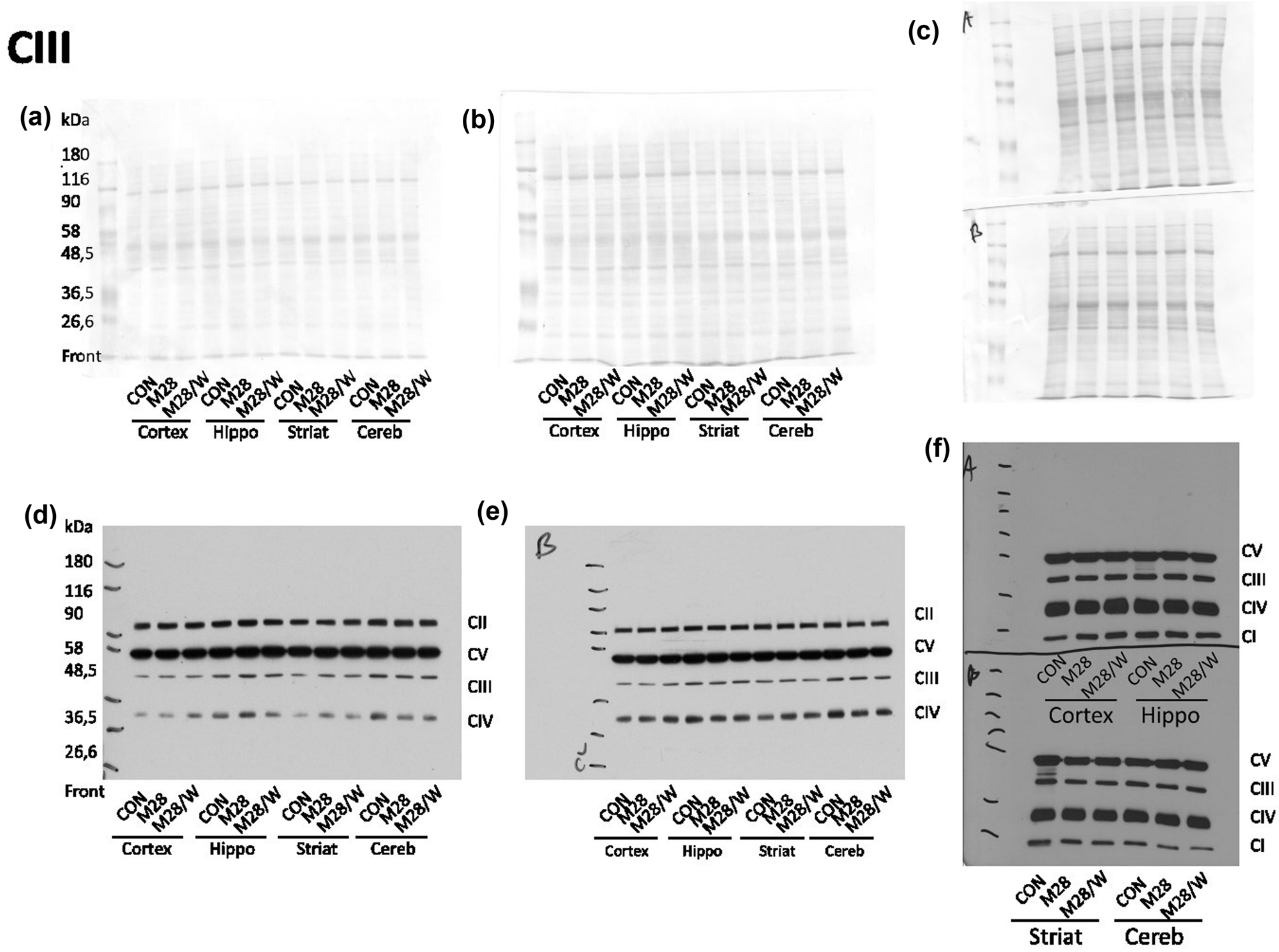

Figure S3: Typical Ponceau S staining and Western blots for detection of complex III. Proteins (10 μg protein per lane) in samples from cortex, hippocampus (Hippo), striatum (Striat) and cerebellum (Cereb) were separated using SDS-PAGE (10% gel, at constant 200 V for 45 min.) and transferred to a nitrocellulose membrane (at constant voltage 100 V for 90 min.). The total amount of proteins bound to the membranes was stained with Ponceau S (a)–(c). Complex III of the respiratory chain was detected with Total OXPHOS Rodent WB antibody diluted 1:50,000 (d)–(f).

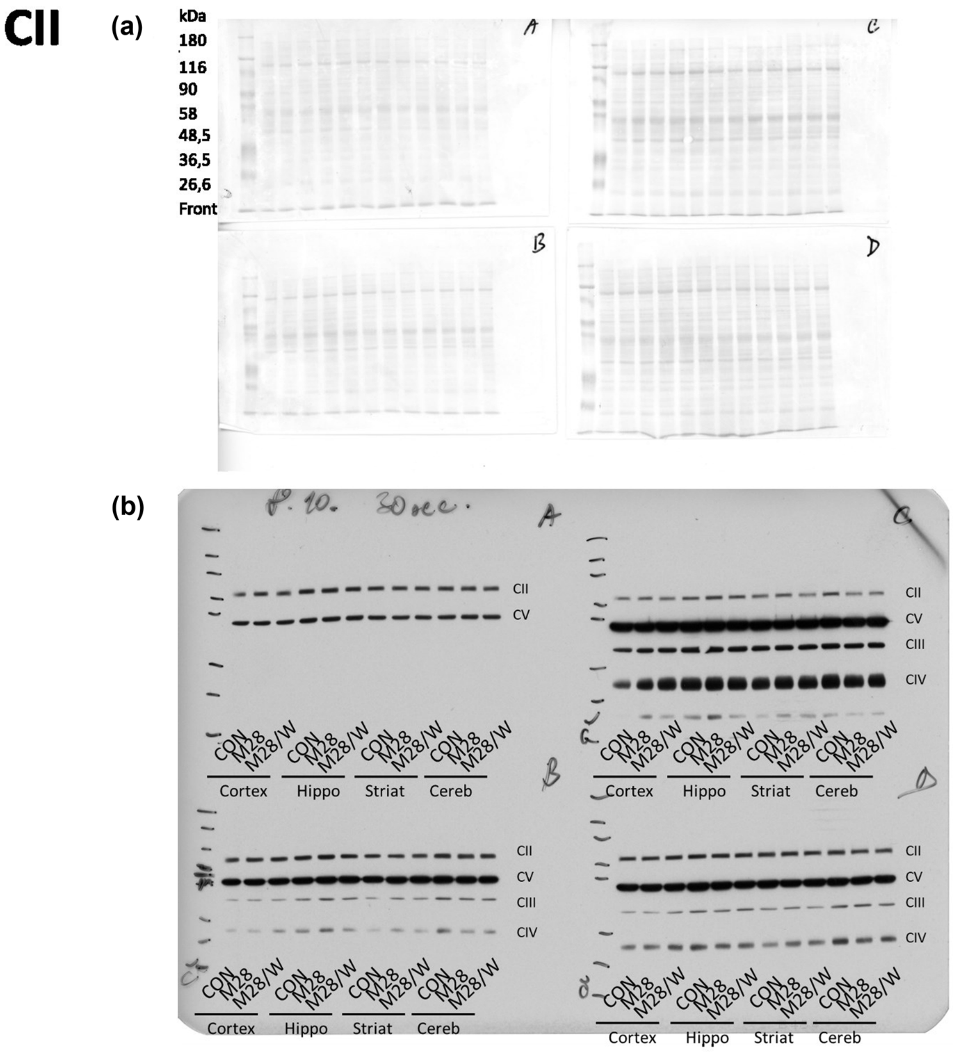

Figure S4: Typical Ponceau S staining and Western blots for detection of complex II. Proteins (10 µg protein per lane) in samples from cortex, hippocampus (Hippo), striatum (Striat) and cerebellum (Cereb) were separated using SDS-PAGE (10% gel, at constant 200 V for 45 min.) and transferred to a nitrocellulose membrane (at constant voltage 100 V for 90 min.). The total amount of proteins bound to the membranes was stained with Ponceau S (a). Complex II of the respiratory chain was detected with Total OXPHOS Rodent WB antibody diluted 1:50,000 (B).

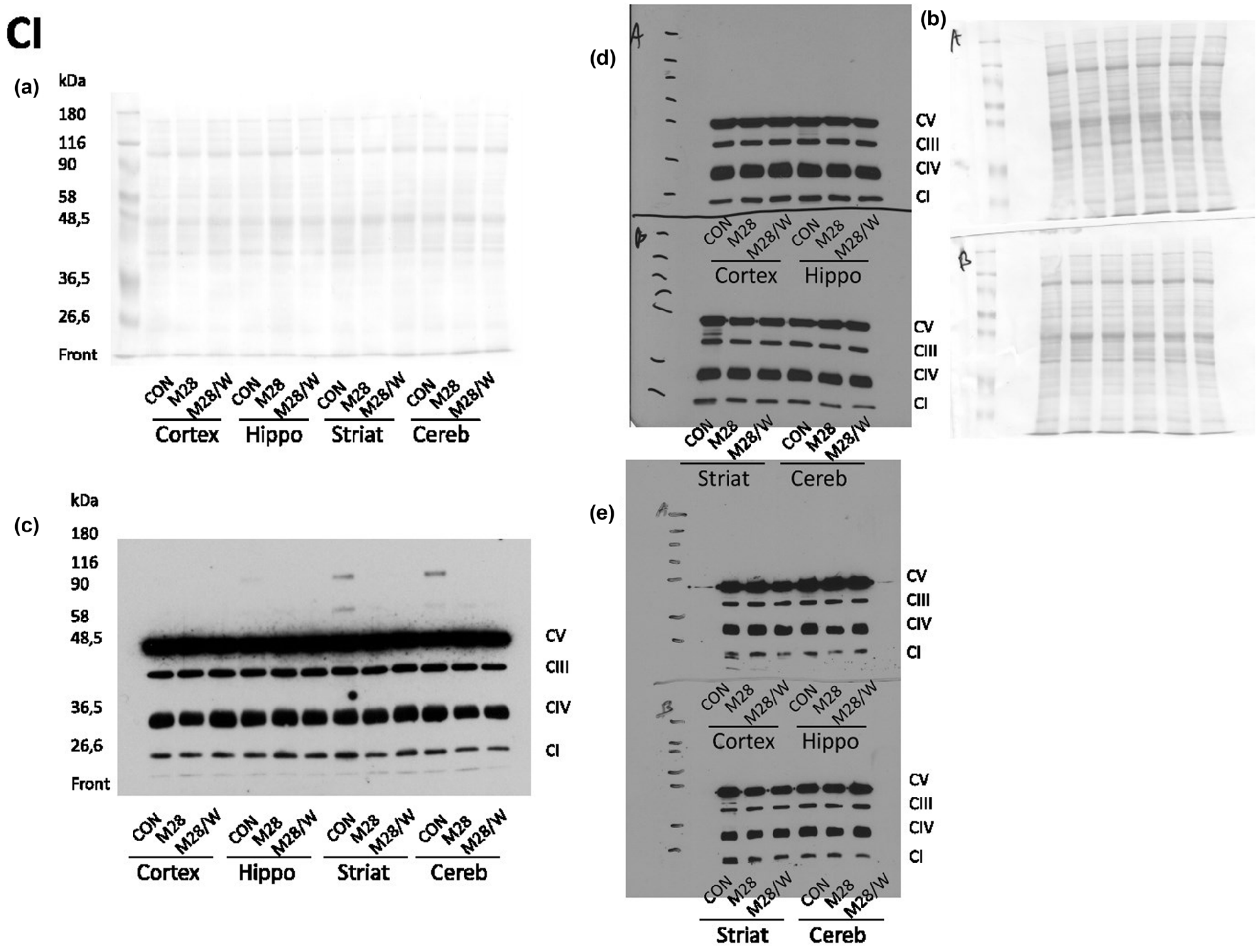

Figure S5: Typical Ponceau S staining and Western blots for detection of complex I. Proteins (10 µg protein per lane) in samples from cortex, hippocampus (Hippo), striatum (Striat) and cerebellum (Cereb) were separated using SDS-PAGE (10% gel, at constant 200 V for 45 min.) and transferred to a nitrocellulose membrane (at constant voltage 100 V for 90 min.). The total amount of proteins bound to the membranes was stained with Ponceau S (a) and (b). Complex I of the respiratory chain was detected with Total OXPHOS Rodent WB antibody diluted 1:10,000 (c)–(e).
